# Identification of Long-Distance Transmissible mRNA between Scion and Rootstock in Cucurbit Seedling Heterografts

**DOI:** 10.3390/ijms21155253

**Published:** 2020-07-24

**Authors:** Wenqian Liu, Chenggang Xiang, Xiaojun Li, Tao Wang, Xiaohong Lu, Zixi Liu, Lihong Gao, Wenna Zhang

**Affiliations:** 1 Beijing Key Laboratory of Growth and Developmental Regulation for Protected Vegetable Crops, China Agricultural University, Beijing 100193, China; lwq08@cau.edu.cn (W.L.); ChenggangXiang0527@outlook.com (C.X.); S20183172263@cau.edu.cn (X.L.); B20183170825@cau.edu.cn (T.W.); luxh0317@gmail.com (X.L.); 2016317010308@cau.edu.cn (Z.L.); 2 College of Life Science and Technology, HongHe University, Mengzi 661100, China

**Keywords:** cucurbit, grafting, long-distance signal, mRNA movement

## Abstract

Grafting has been widely used to improve plant growth and tolerance in crop production, as well as for clarifying systemic mRNA signaling from donor to recipient tissues in organ-to-organ communication. In this study, we investigated graft partner interaction mechanisms of *Cucumis sativus* (Csa) and *Cucurbita moschata* (Cmo) using a large-scale endogenous mRNA transport. The results indicated that most mobile transcripts followed an allocation pathway from source to sink. Gene ontology (GO) enrichment and Kyoto Encyclopedia of Genes and Genomes (KEGG) analysis showed that mRNA mobility functions are universally common and individually specific. Identification of mRNA mobility between distant tissues in heterografts with RT-PCR (reverse transcription PCR), RT-qPCR (reverse transcriptional quantitative real time PCR), and clone sequencing were used to estimate 78.75% of selected mobile transcripts. Integration of bioinformatic analysis and RT-qPCR identification allowed us to hypothesize a scion-to-rootstock-to-scion feedback signal loop of Csa move-down and Cmo move-up mRNAs, where Csa scion move-down mRNAs were involved in carbon fixation and biosynthesis of amino acid pathways, and Cmo root received Csa move-down mRNA and then delivered the corresponding Cmo upward mRNA to scion to improve photosynthesis of cucumber scion. This formed a feedback signal loop of scion-to-rootstock-to scion to explain why pumpkin rootstock enhanced cucumber production in the industry, which was utilized for organ communication and mediates photosynthesis processes in heterograft cucurbit crops.

## 1. Introduction

Grafting has been widely used in the propagation and production of most horticultural plant species to enhance growth vigor and adapt to stressful environmental conditions. Grafting can improve tolerance to salinity, alkalinity, drought, and extreme temperature stress, as well as enhance nutrient uptake [1,2]. It is well documented that communication or interaction exists between plant heterologous organs [3,4,5]. Systemic exchanged signals, which include water, minerals, hormones, photosynthetic assimilation products, amino acids, proteins, and RNAs, are delivered by the vasculature of the xylem and phloem systems [6].

Phloem has long been known to facilitate long-distance signaling from source to sink in association with companion cells and parenchyma [7]. Phloem-delivered macromolecules contain numerous non-cell autonomous proteins and RNAs, including small RNAs, non-coding RNAs, and mRNAs [8,9]. Mobile mRNAs have an effect on development in their destination tissues, and some mRNAs encoding transcriptional non-cell autonomous proteins move through plasmodesmata (PD), regulating meristem development, leaf shape, fruit size, tuberization, and root architectures after translation [10,11,12,13,14]. Grafting was an important tool in these previous studies for discovering how long-distance signaling coordinates and modifies plant development in response to endogenous and environmental conditions. Thus, characterization of mRNA transport, and how mRNAs physiologically function between heterologous organs, is important for fully understanding the impact of grafting.

Many efforts have been made to address the mechanism by which transcribed mRNAs in companion cells are trafficked into sieve elements through the interconnecting PD. RNA molecules, as components of various long-distance signaling systems, were shown to first interact with RNA-binding proteins and then form Ribonucleoprotein (RNP) complexes [14]. These RNP complexes play significant roles in determining the process and fate of RNAs in living cells [15,16,17]. In addition, studies of individual RNAs have identified specific sequence motifs such as CUCUU, conserved stem-loop structures (e.g., viroid), tRNA-like structures [18,19,20] and 5-methylcytosine [21] that are necessary for RNA entry into the sieve tube system [14,22]. However, a lack of available knockout mutants for coordinate genes remains a challenge in addressing the biological function of potential mobile mRNAs.

Numerous studies have reported that mRNAs exchange in heterografted systems, such as the parasitic plant *Cuscuta pentagona*, *Arabidopsis/Nicotiana benthamiana*, grapevine (*Vitis vinifera*), *Arabidopsis* ecotypes, cucumber (*Cucumis sativus*)/watermelon (*Citrullus lanatus*), *Nicotiana benthamiana*, tomato (*Solanum lycopersicum*), and pumpkin (*Cucurbita maxima*)/melon (*Cucumis melo*) combinations, indicating large amounts of bidirectional mRNA exchanges between the two species/graft partners [23,24,25,26,27,28,29]. Large numbers of mRNAs were found to be directionally moving between diverse organs via the vasculature, but the quantity and directions of mobile mRNA varied under different growth conditions and developmental stages. The detection of both mRNA movements from root-to-shoot by gradient distribution and Pi-stress mRNA signaling in specific organs suggested destination-selective trafficking [25,27]. Previous studies suggested that systemic mobile mRNAs were system specific, but not all functional [27,28,29]. Thus, it is urgent to figure out the function characterization of a large number of mobile mRNAs.

Cucumber (*Cucumis sativus* L.) (Csa) is a major vegetable crop grown under protected cultivation in China. To protect against biotic and abiotic stresses, cucumber seedlings are mainly cultivated by heterografting onto commercial pumpkin (*Cucurbitea moschata*) (Cmo) rootstocks [30,31,32,33,34,35,36]. However, the mechanisms by which grafting alters plant growth and development, especially how mRNA signals exchanged between scion and rootstock affect adaption and tolerance to environmental conditions, are still unknown. In this study, we developed a heterograft system using Csa and Cmo to investigate scion–rootstock mRNA movement. Differences in genomic and transcriptomic information allowed clear discrimination when classifying the origin of transcripts. An analysis of published graft systems combined with our data allowed us to identify mobile transcripts in heterografted cucurbits. Taken together, these mRNAs, being involved in scion-to-rootstock and rootstock-to-scion long-distance transport, played roles in a wide biological and physiological processes of cucurbit crop at the whole-plant level. The aim of this study was to characterize the systemic mRNA communication between scion and stock facilitating biological and physiological processes for further understanding the mechanisms of grafting alters plant growth and development.

## 2. Results

### 2.1. A High Number of Transcripts Directionally Movement in Grafted Cucumber and Pumpkin Seedlings

**Figure 1:**
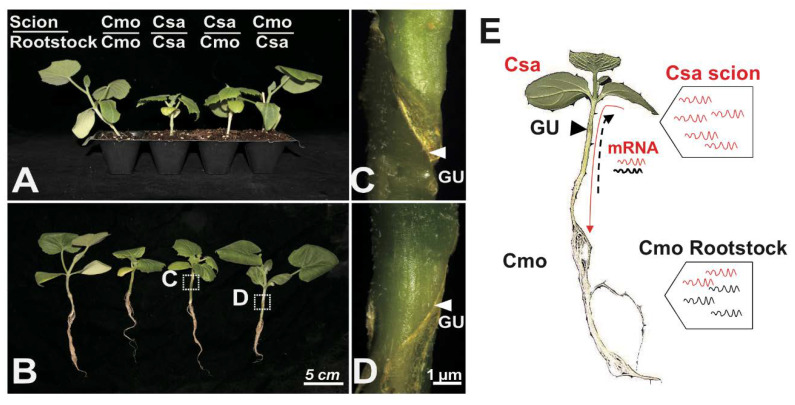
Hypocotyl grafting of *Cucumis sativus* and *Cucurbitea moschata* seedlings. (**A**) *Cucumis sativus* (scion)/*Cucurbitea moschata* (rootstock) (Csa/Cmo) and reverse grafting (Cmo/Csa) heterograft combinations and homografts (Csa/Csa, Cmo/Cmo). Csa and Cmo seedlings of the same size were used for hypocotyl grafting. Grafting was performed when Csa seedlings were approximately 2 cm in diameter with the first true leaf; Cmo seedling carried flattened cotyledons and were 1 cm diameter with first true leaf. The graft junction was healing seven days after grafting. (**B**) Growth of seedlings from four grafting combinations. The whole first true leaf with petiole and whole root of all grafts were harvested for further RNA-seq determination. To avoid individual bias, each graft combination included 8–9 individual grafted plants. Graft-union magnification of (**C**) Csa/Cmo and (**D**) Cmo/Csa seedlings shown in (**B**); arrowheads indicate graft union (GU). (**E**). Scion-to-rootstock long-distance movement of heterologous mRNAs through the phloem was identified in hypocotyl-grafted cucurbits. If Csa transcripts (red) moved to Cmo stock across the grafting union, they could be distinguished from Cmo transcripts (black) based on genomic single nucleotide polymorphism (SNP) differences. The first true leaf and whole root were sampled for further analyses. Dotted arrow indicates rootstock-to-scion mobile Cmo transcripts (black) moving to Csa through cell-to-cell spread or a cycle pathway of scion-to-rootstock-to-scion.

To better test the transportability of RNA signaling molecules in heterografted *Cucumis sativus* (scion)/*Cucurbitea moschata* (stock) (Csa/Cmo) and reverse grafting (Cmo/Csa) combinations, we devised a strategy based on size-matched hypocotyl grafting of cucumber and pumpkin seedlings with one true leaf (Figure 1A–D). The first true leaf and whole root of at least nine plants in three biological replicates were harvested and evaluated by genomic PCR for tissue contamination prior to submitting the samples for genomic DNA and RNA sequencing. To ensure there was systemic RNA movement between scion and stock in all samples, RT-PCR with specific primers recognizing the well-known mobile mRNA *Cmo/CsaNACP* were performed as a positive control (Figure S1). In the predicted source-to-sink(s) signaling system, the true leaf of the scion was the donated organ and the rootstock was the recipient. We expected to identify scion-to-rootstock long-distance movement of heterologous mRNAs through the phloem by integration of genomic DNA and RNA-seq data (Figure 1E).

**Figure 2:**
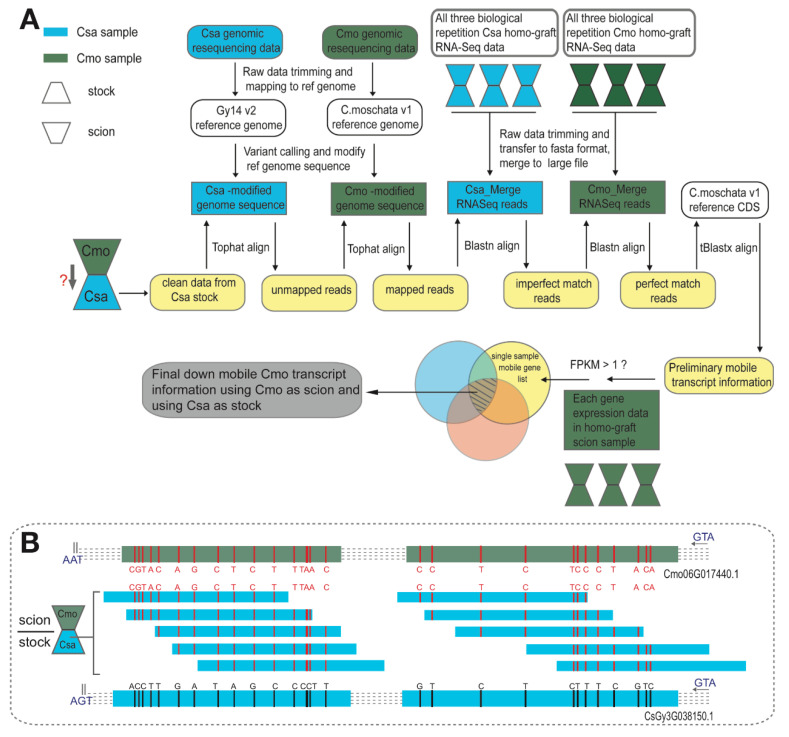
Single Nucleotide Polymorphism (SNP) identification pipeline and detection of mobile RNA. (**A**) Schematic of bioinformatics pipeline used for processing RNA-Seq and DNA-Seq data obtained from *Cucumis sativus* (Csa) and *Cucurbitea moschata* (Cmo) heterografts. Pipeline features include genomic resequencing data processing, variant calling, and mobile transcript detection. Data obtained from Csa samples are colored in blue hues and data from Cmo in dark green hues. Individual pipeline steps are described in the Materials and Methods Section. (**B**) Example read count frequencies for informative SNPs of predicted scion mobile transcripts in stock. Several reads of one transcript coming from Csa stock (blue bar in middle) carry two or more diagnostic SNP loci (red lines) after comparing with *C. sativus* transcript CsGy3G038150.1 (blue bar in bottom), but without SNP loci comparing with *C. moschata* transcript Cmo06G017440.1 (green bar with red lines upon).

**Figure 3:**
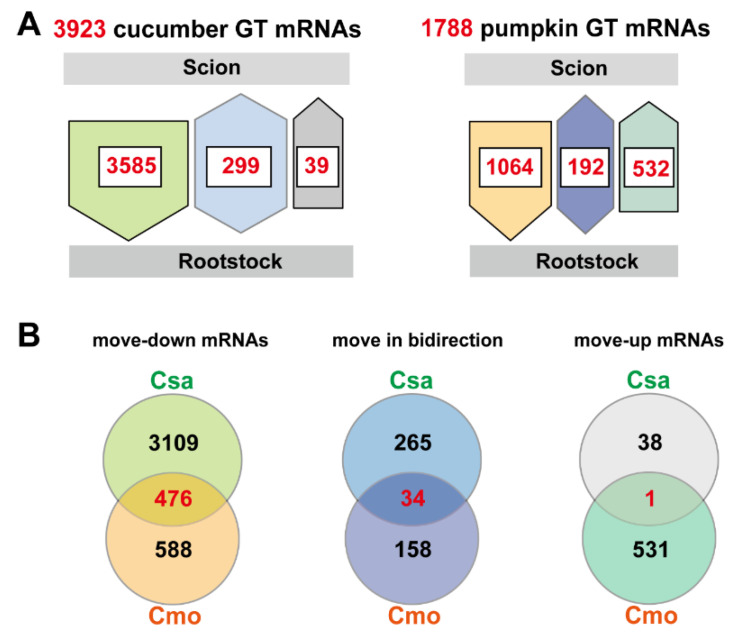
Long-distance movement of heterologous mRNAs between rootstock and scion. (**A**). Scheme with numbers and direction of mobile RNAs exchanged between heterologous tissues and exchanged between species. Nine transcript mobility classes were identified between shoot and root tissues and Csa and Cmo species. Three different possibilities of movement were found under each variable: (i) unidirectional rootstock-to-scion; (ii) unidirectional scion-to-rootstock; and (iii) bidirectional movement. GT, graft-transmissible. (**B**). Venn diagrams overlapping after homology analysis of cucumber and pumpkin in all directions. To compare Csa and Cmo homologous mRNAs, 1788 Cmo mRNAs matching 1781 homologous Csa protein-encoding mobile mRNAs by orthology analysis with best hits were used in the study. After filtering duplications, 1561 homologous Csa transcripts representing Cmo mobile transcripts were used for further analysis. Csa, *Cucumis sativus*; Cmo, *Cucurbita moschata*.

To analyze graft-transmissible Csa and Cmo transcripts present in the recipient heterografted tissues, the following conditions were applied: a mobile transcript had to be detected at least twice within three biological replicates. The mobile transcripts identified in rootstock and scion samples from reciprocally grafted seedlings allowed us to trace the direction of movement into heterologous tissues and species dependency (Figure 2A,B). Furthermore, we classified the directionality of the identified mobile mRNAs into three categories by comparing Cmo mRNAs found in Csa scion grafted on Cmo rootstock with the reverse grafting combination. Based on these criteria, we were able to unambiguously assign 3923 Csa and 1788 Cmo transcripts. In 3923 Csa mRNAs, 91.4% (*n* = 3585) Csa transcripts presented a unidirectional migration from Csa scion to Cmo rootstock, 0.99% (*n* = 39) of them migrated from Cmo rootstock to Csa scion in unique direction, and 7.6% (*n* = 299) of them were bidirectional movement. Likewise, 59.5% (*n* = 1064) Cmo transcripts migrated from Cmo scion to Csa rootstock and 10.7% (*n* = 192) of them migrated from Cmo rootstock to Csa scion in both directions. These results match the major flow direction of sucrose in the phloem from source leaves to roots in hypocotyl-grafted seedlings. Compared to move-up Csa mRNAs, more Cmo mRNAs were found to be able to migrate from Cmo rootstock to Csa (Figure 3A).

### 2.2. Function Characterization of Mobile mRNAs of Cucumber and Pumpkin Are Universal Common

To compare Csa and Cmo homologous mRNAs, 1788 Cmo mRNAs matching 1781 homologous Csa protein-encoding mobile mRNAs by orthology analysis with best hits were used in the study. After filtering duplications, 1561 homologous Csa transcripts representing Cmo mobile transcripts were used for further analysis (Table S1). By homologous BLASTP analysis with Csa transcripts, 476, 34, and 1 overlapped cucurbit transcripts were found to migrate from scion to rootstock, move in bidirectional, and migrate from rootstock to scion, respectively (Figure 3B and Table S2).

**Figure 4:**
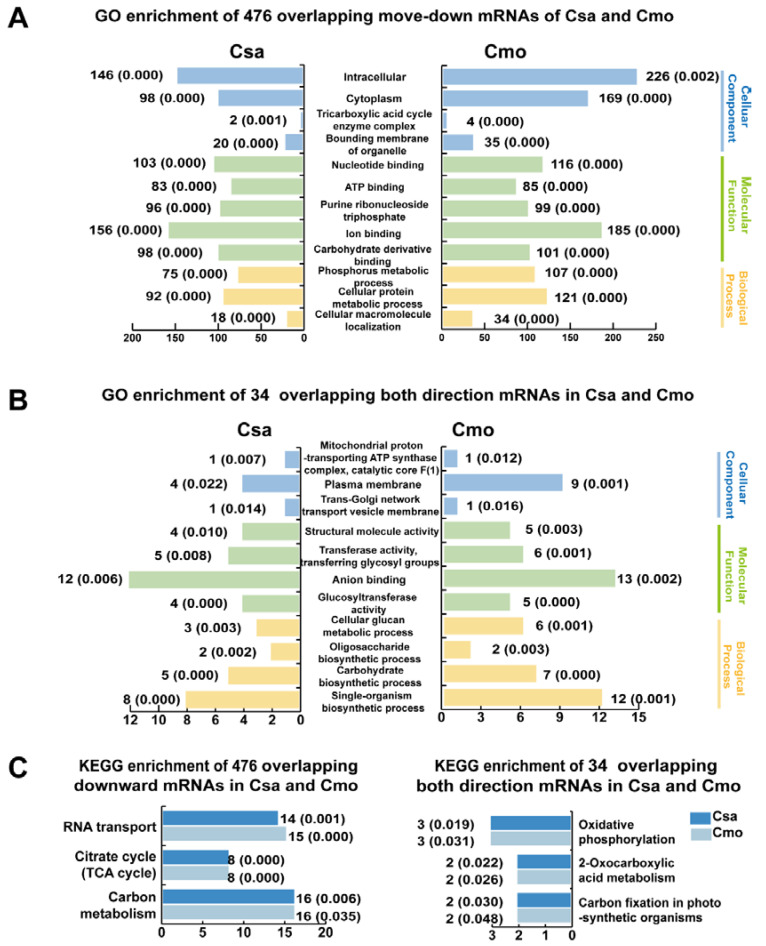
GO enrichment and KEGG pathway annotation of overlapping mobile mRNAs in all directions. (**A**,**B**) GO enrichment and (**C**) KEGG pathway analysis of overlapping mobile mRNAs in different directions. GO enrichment and KEGG pathway analysis was performed using the OmicShare tools, a free online platform for data analysis (http://www.omicshare.com/tools). Graphs were generated by EXCEL (Office 2010) and colored graphs indicate the classifications of molecular function, cellular component, and biological process. The numbers beside each graph represent the number of genes in the cluster and the *p* value.

Gene Ontology (GO) and Kyoto Encyclopedia of Genes and Genomes (KEGG) enrichment analysis of these mobile mRNAs were performed to determine the function classification. Firstly, in 476 overlapping move-down mRNAs of Csa and Cmo, intracellular and cytoplasm of cellular component, nucleotide binding, ATP binding and ion binding of molecular function, and cellular macromolecule localization of biological process were main groups of GO classification (Figure 4A). Those 476 move-down mRNAs of Csa and Cmo were mainly enriched in RNA transport, citrate cycle and carbon metabolism KEGG pathways (Figure 4C). In 34 overlapping move bidirectional mRNAs of Csa and Cmo, mitochondrial proton transporting ATP synthase complex, plasma membrane of cellular component, glucosyltransferase activity, anion binding of molecular function, and carbohydrate biosynthetic process, cellular glucan metabolic process of biological process were main groups of classification (Figure 4B). They were mainly enriched in oxidative phosphorylation, 2-oxocarboxylic acid metabolism and carbon fixation KEGG pathways (Figure 4C). In move-up mRNAs, only one candidate was identified moving upward with overlap in Csa and Cmo mRNAs. CmoCh15G008030 encoded a 2-succinylbenzoate-CoA ligase and the homologous Csa mRNA, CsGy5G013370 encoding an acyl-CoA synthetase were identified mobile in upward direction (Figure 3B and Table S2).

**Figure 5:**
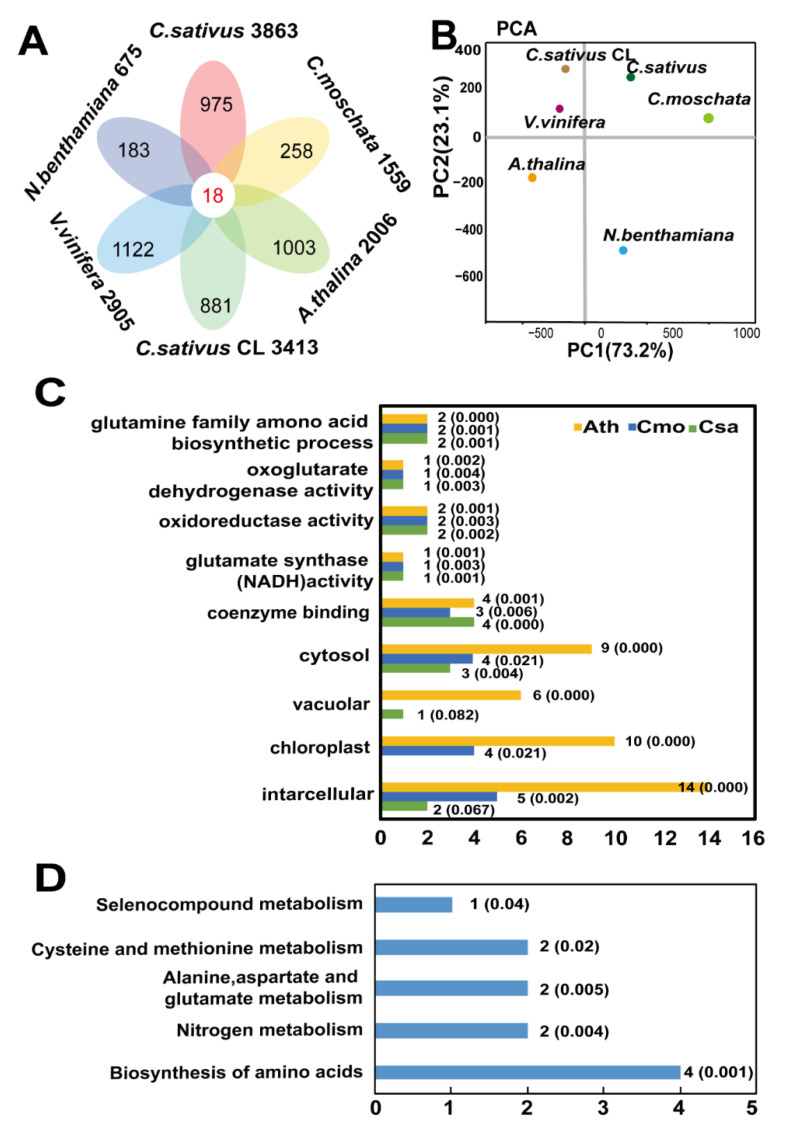
Characterization of orthologous mobile mRNAs identified in different heterograft systems. (**A**) Venn diagram of orthologous mobile mRNAs identified in all six heterograft systems. Mobile mRNAs orthologs in all systems were based on the best reciprocal BLASTP hits gene ID and annotation. (**B**) Four parameters included percentage of mobile mRNAs, percentage of nonmobile mRNAs, CDS length of mobile mRNAs, and CDS length of nonmobile mRNAs were analyzed by EXCEL (Office 2010) in all different species (Table S10). PCA was performed with four parameters by online Omicshare PCA tools. X-axis represents the contribution rate of first principal component and Y-axis represents the contribution principal component. Points represent each sample class. (**C**) Top GO enrichment of 18 common mobile transcripts in Csa, Cmo, and *A. thaliana*. Numbers indicate number of genes. (**D**) Top KEGG pathways of 18 common mobile transcripts in Csa, Cmo, and *A. thaliana*. Numbers indicate number of genes and the *p* values. Graphs were generated by EXCEL (Office 2010). Csa, *Cucumis sativus*; Cmo, *Cucurbita moschata*; Ath, *Arabidopsis thaliana*.

To understand mobile mRNAs in different species shared common characteristics, we identified the orthologous genes in databases containing predicted or identified phloem-delivered long-distance mRNAs in *A. thaliana*, grape, cucumber heterografted with watermelon, and *N. benthamiana* heterograft systems [24,25,26,27]. Through homologous BLASTP analysis, a core group of 18 mobile mRNAs was found to exist in various systems of *A. thaliana* (*n* = 1992), grapevine (*V. vinifera*) (*n* = 3333, 3175 of them corresponding to 2905 *A. thaliana* genes), cucumber heterografted with watermelon (*Cucumis sativus* CL) (*n* = 3546, 3415 of them corresponding to 3083 orthological *A. thaliana* genes), Csa (*Cucumis sativus*) (*n* = 3923, 3863 of them corresponding to 3525 orthological *A. thaliana* genes), Cmo (*Cucurbitea moschata*) (*n* = 1788, 1780 of them corresponding to 1536 orthological *Arabidopsis* genes), and *N. benthamiana* heterograft (*n* = 1164, 1085 of them corresponding to 716 orthological *A. thaliana* genes) mobile transcripts (Figure 5A, Tables S3 and S4). Principal Component Analysis (PCA) suggested that all six species maintained an evolutionary relationship and were able to be distinguished in PC1 (Figure 5B, Table S10). Those mRNAs were involved in broad biological processes, such as intracellular, cytosol of cellular component, coenzyme binding, glutamate synthase, oxidoreductase activity, and oxogluatrate dehydrogenase activity of molecular function, and glutamine family amino acid biosynthetic process (Figure 5C). Those 18 overlapping mRNAs were enriched in biosynthesis of amino acids, nitrogen metabolism, and methionine metabolism pathways (Figure 5D). GO and KEGG classification indicated that mobile mRNAs of cucumber and pumpkin are function classified with overlaps.

### 2.3. Function Characterization of Mobile mRNAs of Cucumber and Pumpkin Are Individually Different

**Figure 6:**
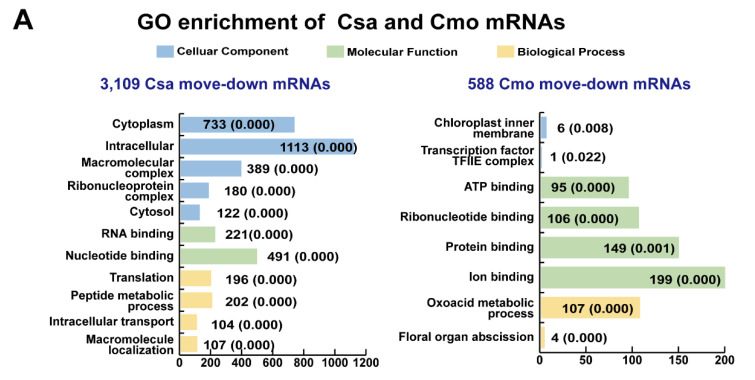

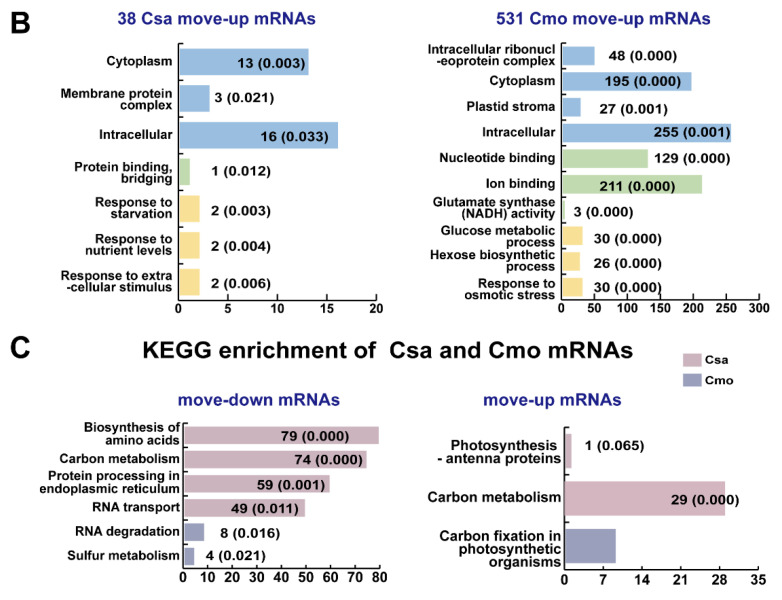
GO enrichment and KEGG pathway annotation of overlapping mobile mRNAs in upward and downward directions. (**A**,**B**) GO enrichment and (**C**). KEGG pathway analysis of upward and downward mobile mRNAs in all directions. Colored graphs indicate the classifications of molecular function, cellular component and biological process. Graphs were generated by EXCEL (Office 2010). The numbers beside each graph represent the number of genes in the cluster and the *p* value. Csa, *Cucumis sativus;* Cmo, *Cucurbita moschata*.

Most Csa and Cmo mobile mRNAs presented individual characterizations. In total, 3109 move-down Csa mRNAs were mainly enriched in intracellular, macromolecular, and ribonucleoprotein complex of cellular components, nucleotide binding and translation, peptide metabolic, intracellular transport, and macromolecule localization molecular functions (Figure 6A and Table S5). They were mainly enriched in biosynthesis of amino acids, carbon metabolism, and protein processing in endoplasmic reticulum KEGG pathways (Figure 6C and Table S5). The 588 move-down Cmo mRNAs were mainly enriched in chloroplast inner membrane and transcription factor TFIIE complex of cellular component, ribonucleotide binding, protein binding and ion binding of molecular functions, oxoacid metabolic process, and floral organ abscission of biological process. They mainly gathered in RNA degradation and sulfur metabolism KEGG pathways (Figure 6C and Table S6). The 38 upwards Csa mRNAs were enriched in cytoplasm, membrane protein complex, intracellular of cellular components, protein binding and bridging of molecular functions, response to starvation, nutrient levels, and extracellular stimulus of biological processes. They were mainly enriched in photosynthesis antenna proteins KEGG pathway (Figure 6B and Table S5). The 531 upwards Cmo mRNAs were enriched in intracellular, ribonucleoprotein complex, cytoplasm, plastid stroma and intracellular components of cellular components, nucleotide and ion binding functions of molecular functions, glutamate synthase (NADH) activity, glucose metabolic process, hexose biosynthetic process, and response to osmotic stress of biological processes (Figure 6B and Table S5). They were mainly enriched in carbon metabolism and carbon fixation in photosynthetic organisms KEGG pathway (Figure 6C and Table S6).

### 2.4. Identification of Long-Distance Trafficking Between Rootstock and Scion of Csa/Cmo mRNA

**Figure 7:**
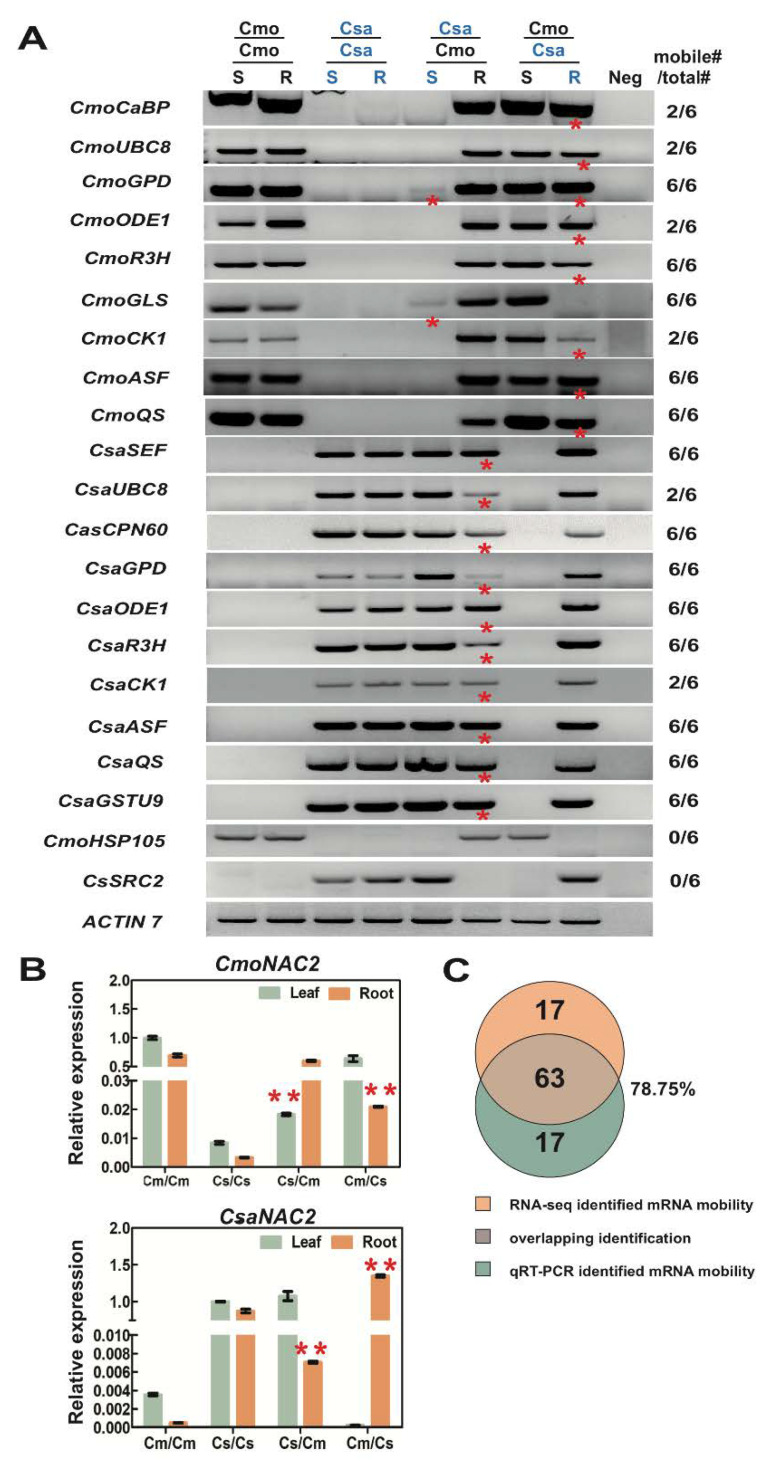
Identification of Csa and Cmo mobile transcripts using RT-PCR and RT-qPCR in heterograft systems. (**A**) RT-PCR assays with specific primers confirm *CmoCaBP*, *CmoUBC8*, *CmoGPD*, *CmoODE1*, *CmoR3H*, *CmoGLS*, *CmoCK1*, *CmoASF*, *CmoQS*, *CsaSEF*, *CsaUBC8*, *CsaCPN60*, *CsaGPD*, *CsaODE1*, *CsaR3H*, *CsaCK1*, *CsaASF*, *CsaQS,* and *CsaGSTU9* underwent either unique or double directional movement in heterografts. *CmoHSP105* and *CsaSRC2* were not detected in the mobile mRNA list. Each graft combination was divided into six heterograft pool replicates, and each pool included 6–9 individual grafted plants. Red asterisks indicate the direction of mRNA transport, and the ratio numbers indicate mRNA mobility detected in sample repetitions by RT-PCR. Csa, *Cucumis sativus*; Cmo, *Cucurbita moschata*; S, shoot; R, root; Neg, negative. (**B**) RT-qPCR analysis of Csa/CmoACTIN7 as an internal reference was conducted on heterograft Cmo and Csa samples. Specific primers for RT-qPCR were verified to only recognize *CmoNAC2* transcripts, and did not amplify *CsaNAC2* transcripts to any significant extent. A significant increase in the Csa root of Cmo/Csa heterografts allowed us to determine potential Cmo mRNAs’ downward movement. Likewise, *CsaNAC2* transcript downward mobility was identified in the same way. Cs, *Cucumis sativus*; Cm, *Cucurbita moschata*. Red asterisks indicate significant differences (<0.001) amongst the same tissue of homograft controls using *t*-test for independence of variables in a contingency table. Biological replicates: *n* > 9. Graphs were generated by GraphPad Prism5.0. Error bars indicate standard deviation. (**C**). Venn map indicates a correlation (78.75%) between RNAseq and RT-qPCR identification assays. Eighty mobile mRNAs were selected for testing, and 63 of them matched mobility identified by RT-qPCR.

To test whether mRNA molecules move long distance between rootstock and scion, we performed RT-PCR assays on RNA samples of all homo- and heterograft combinations using primers designed to recognize specific sequences of Csa/Cmo transcripts. Using this approach, we established that the predicted endogenous *CsaCK1* and *CmoCK1* had indeed moved from Cmo scion to Csa rootstock as previously reported. Downward movement of *CmoCaBP* and *CmoASF* was opposite to that predicted from the deep-seq results; *CmoGLS*, *CmoUBC8*, *CmoODE1*, *CmoQS*, and *CmoR3H* showed downward mobility and *CmoGPD* showed dual-direction mobility, which was identical to the deep-seq results. We also identified homologous mRNA mobility in Csa: *CsaUBC8*, *CsaGPD, CsaASF*, *CsaQS*, *CsaGSTU9* all showed a downward movement and *CsaSEF* and *CsaCPN60*, which have bidirectional mobility, were confirmed only to show downward mobility. *CmoHSP105* and *CsaSRC2,* which were absent from the mobile list, were identified not to be mobile as predicted (Figure 7A). The amplification of the proper mobile transcripts was confirmed by subsequent cloning of PCR products into a plasmid vector and sequencing (Figure S2).

**Figure 8:**
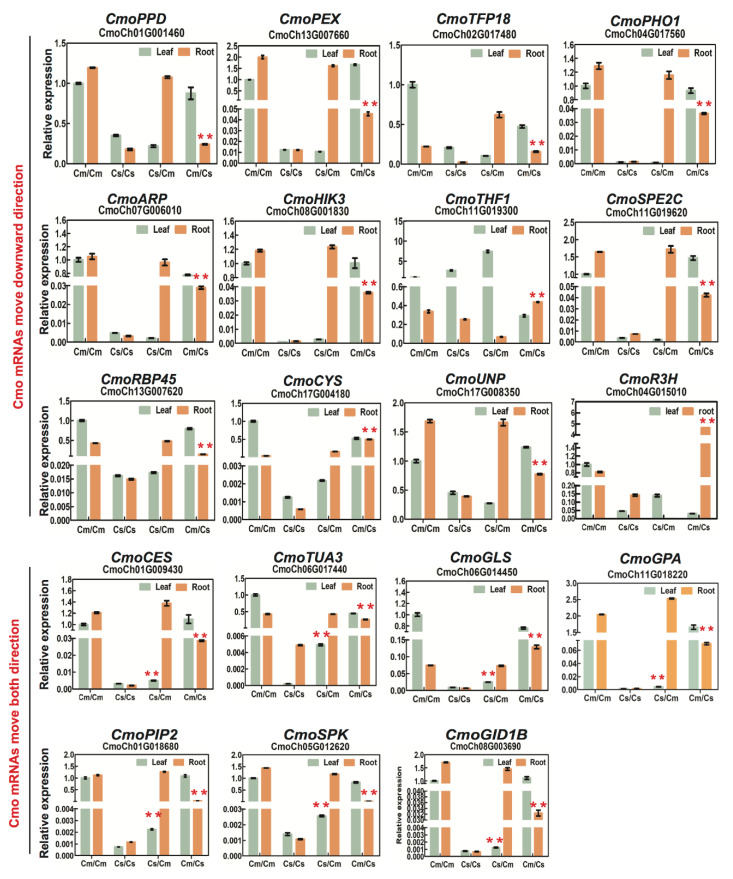
RT-qPCR verification of selected *Csa* mRNA mobility and movement direction in heterografts. *Csa/CmoACTIN7* was used as an internal reference for heterograft Cmo and Csa samples. Specific primers recognizing *Csa* transcripts were used for RT-qPCR, which identified an extremely low mRNA abundance in control Cmo/Cmo homografts. A significant increase in mRNA abundance in Csa/Cmo root and Cmo/Csa shoot suggested potential downward and upward bidirectional movement of Csa mRNA. Graphs were generated by GraphPad Prism5.0. Red asterisks indicate highly significant differences from the same tissue of homograft controls using *t*-test for independence of variables in a contingency table. Cs, *Cucumis sativus*; Cm, *Cucurbita moschata*.

**Figure 9:**
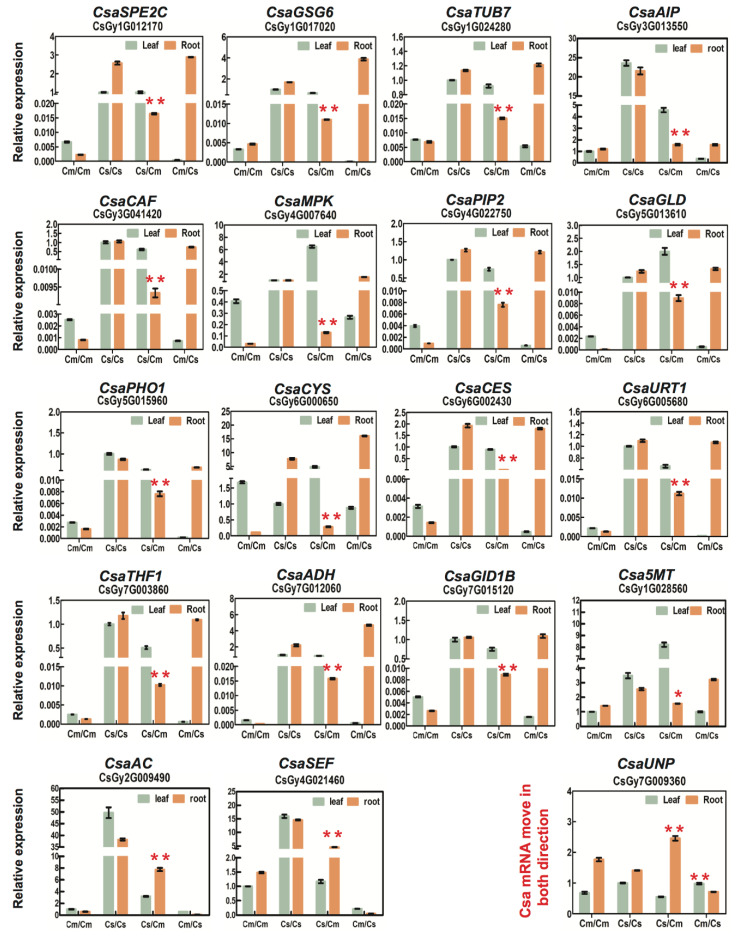
RT-qPCR verification of selected Cmo mRNA mobility and movement direction in heterografts. Specific primers recognizing Cmo transcripts were used for RT-qPCR, which identified an extremely low mRNA abundance in control Csa/Csa homografts. A significant increase in mRNA abundance either in Csa/Cmo shoot or Cmo/Csa root suggested potential downward, upward, or bidirectional movement of Cmo mRNA. Graphs were generated by GraphPad Prism5.0. Red asterisks indicate highly significant differences from the same tissue of homograft controls using *t*-test for independence of variables in a contingency table. Biological replicates: *n* > 9. Error bars indicate standard deviation. Cs, *Cucumis sativus*; Cm, *Cucurbita moschata*.

It is challenging to identify mRNA mobility in heterografts by quantitative PCR (RT-qPCR) because the mRNA must be distinguished from homologous genes using RT-qPCR primers. Specific primers were designed avoiding high sequence identity between Csa and Cmo samples for efficient PCR reactions to amplify specific products in homo- and heterograft samples (Figure S3). To identify mobility of Cmo mRNAs, we need to ensure specific primers recognizing Cmo transcripts but rarely amplifying Csa transcripts for RT-qPCR. We then compared Cmo mRNA abundance of heterografts with that in the same tissue of controls, and a significant increase either in leaf of Csa/Cmo or root of Cmo/Csa allowed us to identify potential hetero-tissue-induced Cmo movement. By the same method, Csa mRNA mobility was identified. Thus, gene-specific signals were successfully detected for selected genes (Figure 7B, Figure 8 and Figure 9). These experiments confirmed the ability of our experimental workflow to identify mobile transcripts. An accurate quantification of the mobile transcripts was not possible, but we estimated a correlation (78.75%, *n* = 80) between transcripts identified by RT-qPCR and those identified by RNA-seq data (Figure 7C and Table S7).

In addition, we found that some transcripts with high abundance of RPKM could not be identified as mobile, such as *CmoCaBP* and *CsaSRC2*, while transcripts with low abundance of RPKM were identified as mobile, such as *CmoPHO1* and *CsaADH* (Table S8). Neither identification result indicates the regular direction of mRNA movement. Thus, we concluded that the mobility direction of those transcripts was not correlated to RNA abundance.

## 3. Discussion

Mobile mRNAs are participating in wide biological processes of plant growth and development. It has been demonstrated that some phloem-mobile mRNAs play physiological roles in recipient sink organs, such as young leaves, roots, and tubers [11,12,24,37,38]. However, the identification of large numbers of mobile mRNAs (hundreds to thousands) from recent plant–parasite or heterograft experimental systems and the high variation across different systems have raised concerns as to the percentage of these mobile mRNAs associated with physiological functions in target tissues [23,24,25,26,27,28,29]. The quantity and direction of mobile mRNAs indicated that the majority of mRNAs were involved in a scion-to-rootstock movement, which was correlated with the major flow of sucrose in the phloem from source leaves to the root in hypocotyl-grafted Csa/Cmo seedlings (Figure 1). The observed distribution patterns and diminished transfer of rootstock-produced mRNA to scion supported the spread of mRNAs in *A. thaliana* and grape ecotype graft plants over the graft junction [25,26].

**Figure 10:**
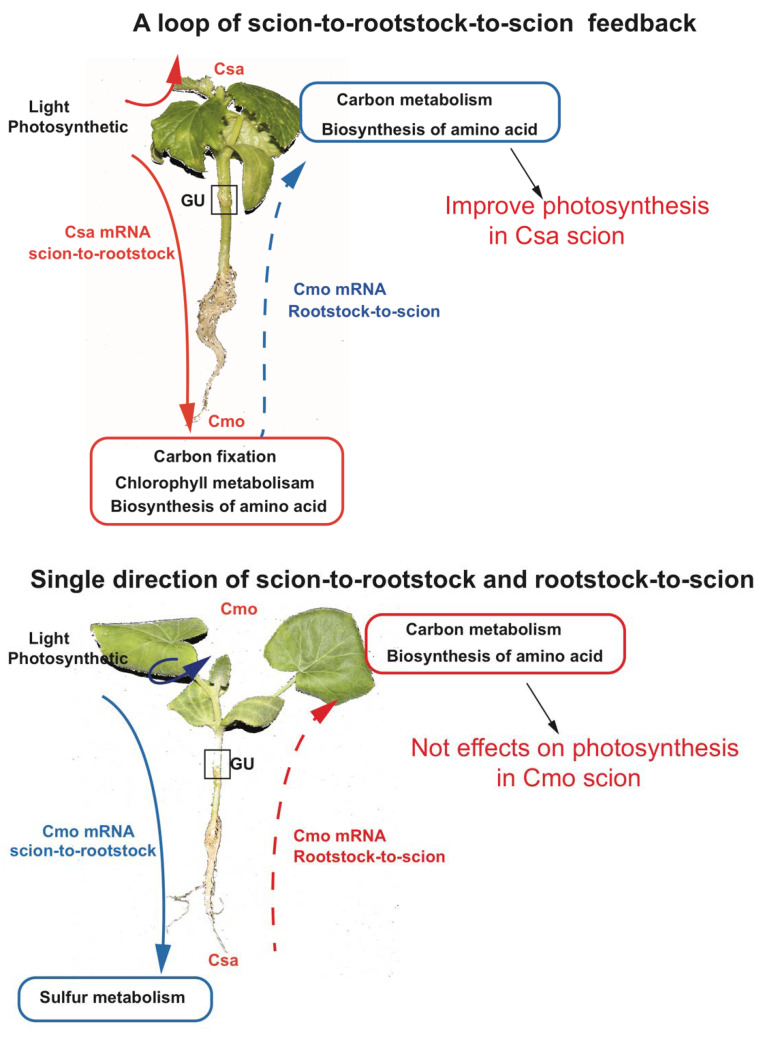
Scheme of Csa and Cmo mRNA movement pathways between pumpkin and cucumber. In the upper panel of cucumber scion grafted onto pumpkin rootstock, the majority of Csa mRNAs relating to photosynthetic are transported into the root of pumpkin, following the major flow of sucrose in the phloem from source leaves to the root in hypocotyl-grafted Csa/Cmo seedlings. Pumpkin rootstock produced rootstock-to-scion axis Cmo mRNAs related to carbon metabolism, chlorophyll metabolism, and amino acid synthesis are migrating into shoot tissues by unknown mechanism or cell-to-cell spreading after grafting union of young seedlings (a few centimeters), thereby increasing cucumber chlorophyll metabolism to enhance photosynthesis. It forms a closed loop of scion-to-rootstock-to-scion feedback. In the lower panel of pumpkin scion grafted onto cucumber rootstock, Cmo mRNAs associated with the photosynthetic produced in pumpkin leaves are transported into cucumber roots; however, cucumber rootstock does not respond correspondingly, thus does not deliver Csa mRNAs to effect on photosynthesis of Cmo scion, representing the single directional pathways of scion-to-rootstock and rootstock-to-scion. Csa, *Cucumis sativus*; Cmo, *Cucurbita moschata*.

GO and KEGG analysis provided overviews for mobile mRNAs functions (Figure 4 and Figure 6 and Tables S5 and S6). In total, 3109 move-down Csa mRNAs and 588 move-down Cmo mRNA were both involved in molecular function of ribonucleotide binding and amino acid metabolic pathway. Interestingly, differing from Cmo mRNAs, certain move-down Csa mRNAs participated in carbon fixation and oxidative and chlorophyll metabolism of KEGG pathways. Thus, we concluded that most Csa mRNAs from scion donor to rootstock recipient to function in carbon fixation in the roots (Figure 10).

However, the observed distribution pattern and transfer of rootstock-produced mRNA to scion supported the notion of the co-existence of phloem and against-phloem transport. This distribution of transcripts along the rootstock-to-scion axis correlated with the pattern of mRNAs in *A. thaliana* ecotype graft plants [26]. Hence, one explanation is perhaps that enough time (seven days after grafting) allowed rootstock-produced mRNA migration into shoot tissues by cell-to-cell spreading after grafting union of young seedlings (a few centimeters). Another possible explanation is that scion-to-rootstock migrating mRNAs were initially unloaded from the phloem in the stele region via plasmodesmata symplastically [16], and then, by an unknown mechanism, mobile mRNAs were transferred to cells (e.g., phloem and/or cortical) and moved to the scion [28,29]. When looking at move-up mRNAs, differing from Csa mRNAs, 531 Cmo mRNAs were enriched in carbon fixation in photosynthetic organ of energy metabolism, and peroxisome of cellular process (Tables S5 and S6). Some metabolites, mRNAs, and proteins were reported to produce in the rootstock spread/transport/migrate via cell-to-cell to the ground of the grafted vegetables [39,40]. Pumpkin rootstocks are widely used in cucumber grafting cultivation due to their strong vigor, including enhancing photosynthesis, keeping stability of plasma membrane, and so on [30,31,32,33,34,35,36]. Considering both carbon metabolism in photosynthesis existing in both Csa move-down and Cmo move-up mRNAs, we assumed that Csa mRNAs relating to carbon fixation produced in cucumber aboveground transport into pumpkin roots via phloem firstly. Pumpkin received the mRNA signals then produced downstream Cmo mRNAs related to carbon metabolism and transport into aboveground to trigger photosynthesis reactions. mRNA mobility identification confirmed our speculation; for example, Csa mRNAs (*CsaGPD*, *CsaODE1*, *CsaCYS*) relating to biosynthesis of amino acids and secondary metabolites were identified to move downward. Cmo mRNAs (*CmoHIK3*, *CmoGID1B*) relating to hormone signal transduction were identified as moving upward (Figure 8 and Figure 9 and Table S8). It was correlated to a feedback loop of scion-to-rootstock-to-scion, as proposed in [41,42,43] (Figure 10).

The function of mobile mRNAs classification has universality and individuality, and plant species and grafting system have important influence on mRNA mobility (Figure 4). However, the characterization of mobility direction of homologous mRNA differed in different graft system; e.g., *CmoGPD* (CmoCh13G008180), a transcript encoding glyceraldehyde-3-phosphate dehydrogenase, was identified to move in both up and down directions in both Csa/Cmo and Cmo/Csa heterografts. However, the homologous gene *CsaGPD* (CsGy1G009330) was identified to only move in the down direction (Figure 6 and Figure S2). *CmoCK1*(CmoCh01G010050) and *CsaCK1*(CsGy5G027230), both transcripts encoding choline kinase, were identified as having move-down in Csa/Cmo and Cmo/Csa heterografts. The homologous mRNA *AtCK1*(At1G71697) was confirmed to have bidirectional mobility in grafted *A. thaliana* [25]. Thus, directional mRNA movement is independent of RNA abundance, RNA motifs, and mRNA length [44]. On the one hand, mRNA movement direction is dependent on the target tissue where the mRNA functions and the biological processes in which they participate. On the other hand, movement direction is determined by the growth conditions of different plant species.

Furthermore, large-scale bioinfomatic analysis is useful for screening systemic mobile mRNA in plants, and has been used in *A. thaliana*, *V. vinifera*, *Cucumis sativus* CL, *N. benthamiana*, and *Solanum lycopersicum* plants [23,25,26,27,28,29]. Undoubtedly, omics data analysis has broadened our understanding of signaling and molecule exchange in different plant species under different environmental conditions. However, due to mathematical errors or individual differences, we need to reconfirm signaling mRNA transport using traditional biological means such as RT-PCR, RT-qPCR, and gene editing, because the correlation coefficient for the relationship between RNA-seq and RT-qPCR indicated differences that need to be verified using multiple methods. Furthermore, due to higher similarity of Csa and Cmo transcripts, it was challenging to design specific primers absolutely recognizing Csa or Cmo transcripts. In our study, we developed an effective RT-qPCR identification method to judge mRNA mobility, which greatly improved the screening process. For example, we designed a specific primer sequence of *CmoPHO1* and performed RT-qPCR on the first true leaves and root tissues of all grafting combinations. The transport and directivity of the *CmoPHO1* mRNA were determined by comparing the relative expression of the corresponding tissues of self-grafted cucumber in the heterogenous grafting combinations. However, deeper analysis on the function of mRNA mobility requires studies using gene editing and transgenic plants in the near future.

## 4. Materials and Methods

### 4.1. Plant Materials, Growth Conditions, and Grafting

Cucumber (*Cucumis sativus* L. “Xintai Mici”, laboratory homozygous material) seeds were soaked in warm, sterilized distilled water (55 °C) for 20 min, followed by room temperature (28 °C) soaking and incubation in a wet towel in a dark chamber at 29 °C. After 1 day of incubation, germinated seeds were sown in 50-hole seedling trays with mix matrix (peat: vermiculite:perlite, 2:1:1, by vol.) and cultured in a growth chamber (relative humidity: 70%; 16 h/8 h light/dark; 28 °C/18 °C day/night; light intensity: 190–600 μmol m^−2^·s^−1^). After approximately 7 days, cucumber seedlings 2-cm in diameter and with their first true leaf were used for grafting.

Pumpkin (*Cucurbita moschata* “Qianglishi”, Shouguang Hongliang Seed Co., Shandong, China) rootstock seeds were soaked and sown 7 days after those of cucumber, and were kept for 2 days in a dark chamber at 29 °C; otherwise, both seedling types were grown under the same conditions until reaching 1-cm diameter and the appearance of the first true leaf.

To facilitate homo-, hetero-, and opposite grafting of Csa and Cmo, hypocotyl-grafting was performed when Csa seedlings were approximately 2 cm in diameter with their first true leaf and Cmo seedlings carrying flattened cotyledons were 1 cm in diameter with their first true leaf. The hypocotyls of Csa and Cmo seedlings were approximately the same size. A graft incision (30° angle) 1 cm under the cotyledons was made using a razor blade. After aligning the graft junctions of the scion and rootstock, these were fixed tightly using a graft clip. Grafted plants in seedling trays were covered and incubated for 3 days in a dark growth chamber (28 °C, relative humidity: 100%), followed by another 4 days with weak light in a growth chamber (28 °C/18 °C day/night; light intensity: 60–100 μmol m^−2^ s^−1^; relative humidity: 100%). Emerging axillary roots at the scion were removed. Seven days after grafting, the establishment of a graft junction was examined after removal of the graft clip. The whole first true leaf with petiol and whole root from all grafts (each graft combination included 8–9 individual grafted plants) were harvested using sterile razor blades, immediately frozen in liquid nitrogen, and stored at −80 °C for RNA extraction.

### 4.2. Genomic DNA Isolation and Genomic Purity Verification

Leaves and root tissue of cucumber and pumpkin seedlings were harvested and ground to powder in a mortar supplied with liquid nitrogen. DNA was extracted using a DNA extraction kit (Huayueyang Biotech, Co., Beijing, China, Cat#0419-50) following the manufacturer’s instructions. The resulting genomic DNA was suspended in distilled H_2_O, and DNA concentration was determined by agarose gel electrophoresis (1%, *w*/*v*, agarose, 1× TAE) and quantified using a NanoDrop ND-2000 (Thermo Scientific, Waltham, MA, United States). Fidelity of the extracted DNA was determined by PCR-based detection with specific primers recognizing cucumber and pumpkin *ACTIN7* (Table S9). Genomic PCR was performed with C1000 TouchTM Thermal Cycler (Bio-Rad, US) according to the 2× T5 Super PCR Mix (TSING KE Co., Beijing, China, TSE005) with 2× T5 Super PCR Mix, 0.5 µM Primer and 50 ng genomic template following reaction: initial denaturation, 1 cycle at 98 °C for 3 min, 30–35 cycles at 98 °C for 10 s, 55–58 °C for 10 s, 72 °C 5 s; dissociation stage, 2 min at 72 °C.

### 4.3. RNA Isolation and cDNA Synthesis

Total RNA of the whole first leaf and root of grafts was extracted using a total RNA isolation kit (Huayueyang Biotech, Co., Beijing, China, Cat#0416-50) following the manufacturer’s instructions. To determine RNA quality and concentration, 1 µL of each RNA sample was analyzed by agarose gel electrophoresis (2%, agarose, 1× TBE) and quantified using a NanoDrop ND-2000 (Thermo Scientific, Waltham, MA, United States).

Reverse transcription reactions were performed using a PrimeScript^TM^ RT reagent kit (Takara Biomedical Technology (Beijing) Co., Beijing, China, Cat#RR047A) following the manufacturer’s instructions with the following steps: total RNA (~2 µg) treated with DNase I was denatured at 70 °C for 10 min in the presence of oligo (dT) primer followed by 5 min annealing incubation at 37 °C prior to the reverse-transcription reaction. RT-PCR was conducted under standard PCR conditions with 40–45 cycles using primers listed in Table S9. RT-PCR was performed according to the 2× T5 Super PCR Mix (TSING KE Co., Beijing, China, TSE005) with 2× T5 Super PCR Mix, 1.5 μM Primer and 2 ng cDNA template following reaction: initial denaturation, 1 cycle at 98 °C for 3 min, 40–42 cycles at 98 °C for 10 s, 60 °C for 10 s, 72 °C 5 s; dissociation stage, 2 min at 72 °C.

### 4.4. Construction and Colony Sequencing

To determine mRNA movement, we carried out T-vector construction with RT-PCR products. Amplified RT-PCR products with primers recognizing both Csa and Cmo genes from grafted Csa/Cmo or Cmo/Csa tissues were purified by AxyPrep DNA Gel Extraction Kit (22218KE1, Axygen, US). Purified RT-PCR products were ligated into pClone007 Blunt Simple Vector (TSING KE Co., Beijing, China, TSV-007BS) following the manual protocol. Ligation products were transformed into DH5α competent cells (TSING KE Co., Beijing, China, TSV-A07) using 42 °C heat shock method. Bacterial was cultured on LB solid medium with 100 mg mL^−1^ ampicillin overnight at 37 °C. Ten to twenty single cultured colonies were sanger sequenced in company (TSING KE Co., Beijing, China). Then, Chromas software (TSING KE Co., Beijing, China, www.tsingke.net) and CLC Sequence Viewer 8 (QIAGEN Aarhus A/S) were used to analyze sequence peaks of RT-PCR products.

### 4.5. RNA and DNA Library Construction and Sequencing

At least nine grafted plants were pooled into one replicate (three total biological replicates) for each graft combination. Pooled RNA (quantity > 10 µg, concentration 1–2 µg µL^−1^) and genomic DNA (quantity > 10 µg, concentration 1–2 µg µL^−1^) extracted from the first leaf and root were subjected to next-generation genome and transcriptome sequencing (DNA- and RNA-seq, respectively). Since the genomes of plant material used in this study were not consistent with published cucurbit genomes, next-generation sequencing libraries were generated and sequenced to obtain paired-end reads with a length of 150 bp on an Illumina HiSeq2500 platform (Oebiotech, Shanghai, China). More than 120 Gb (more than 120×) re-sequenced genomic DNA-seq data from Csa and Cmo were generated.

### 4.6. Consensus Genome Construction

Re-sequenced clean reads of Cmo and Csa were mapped to reference genomes to generate variant site information using BWA [45]. Based on variant site information, the reference genome sequences were modified and used to generate a Cmo-modified genome and a Csa-modified genome using bcftools 1.8–1 software [46].

FastQC and Trim Galore [47] were employed for quality control and adaptor trimming of raw DNA-seq reads. After discarding all low-quality reads, only cleaned reads with more than 100 bp were used for further analysis. RNA-seq data of Csa and Cmo homograft (scion and rootstock from same species) tissues were transferred to fasta format and merged as two large files to be used in further analysis (Csa_merge_RNA-seq_file and Cmo_merge_RNA-seq_file).

### 4.7. Mobile RNA Identification by RNA-seq

Mobile transcripts from the Csa scion were assumed to be identifiable from RNA-seq data in the Cmo stock if the following three conditions were satisfied. (i) A number of Cmo reads corresponding to homologous Csa transcripts should contain at least two or more Single Nucleotide Polymorphism (SNP) loci with reference to the Cmo genome, but without comparison with the Csa genome. (ii) Transmissible Csa reads should not perfectly match the Cmo homograft RNA-seq data, but rather match the Csa homograft reads. (iii) The transmissible Csa gene must be expressed in Csa homograft RNA-seq data.

To identify mobile RNAs transmitted from Csa scion to Cmo stock, clean reads from Cmo stock were first mapped to the modified Csa consensus genome using Tophat v2.2 [48] allowing up to three edit distance. The mapped reads were then discarded and the unmapped reads were mapped to the modified Cmo consensus genome using Tophat2 allowing up to one edit distance. The mapped reads were aligned to the Csa_merge_RNASEQ_file using the BLASTn program; perfect matches without any gaps or SNP sites were excluded and the remaining reads were aligned to the Cmo_merge_RNASEQ_file using the BLASTn program again; in this round, only the perfect matches were retained.

The final remaining reads were compared to the Cmo Coding Sequence (CDS) file using the tBLASTx program with E value set to 10^−40^, and a transmissible gene list was generated after removing duplicate gene IDs from the tBLASTx results. FPKM (fragments per kilobase of exon per million fragments mapped) values of the remaining transmissible genes in Cmo homograft stock tissue were investigated and transmissible genes with FPKM values less than 1 were treated as not accumulated and discarded. The remaining reads were treated as transmissible transcripts. For each biological replicate, the detection procedure was executed completely and only the genes appearing in all three biological replicates were treated as final transmissible genes from Csa scion to Cmo rootstock.

### 4.8. GO (Gene Ontology) Enrichment and KEGG (Kyoto Encyclopedia of Genes and Genomes) Pathway Analysis

GO enrichment and KEGG pathway analysis was performed using the OmicShare tools, a free online platform for data analysis (http://www.omicshare.com/tools).

### 4.9. Orthology Analysis

Complete amino acid sequences of cucumber (*Cucumis sativus* L. “Xintaimici”) were compared with those of *A. thaliana*, grapevine (*Vitis vinifera*), cucumber (*Cucumis sativus* L. “Chinese Long”), *N. benthamiana*/tomato, and pumpkin (*Cucurbitea moschata*) using BLASTP with an E value cutoff of 1 × 10^−5^ and transferring *A. thaliana* gene IDs and annotation. Orthologous pairs were identified based on reciprocal best hits from BLASTP. Orthologous pairs that shared the same *A. thaliana* genes were identified as those present in all six species. Unmatched genes were considered specific genes. The same procedure was used to identify orthologous pairs between cucumber and pumpkin by transferring cucumber gene IDs and annotation. Mobile mRNAs identified from the different heterograft systems were used for comparisons.

### 4.10. Quantitative RT-PCR

Quantitative RT-PCR (RT-qPCR) was performed according to the TB Green method in a 5 µL volume using 4 µg total RNA, 2.5 µL TB Green^TM^ *Premix Ex Taq*^TM^ (Tli RNaseH Plus) (Takara Biomedical Technology (Beijing) Co., Beijing, China, Cat#RR420A), and 0.2 µM forward and reverse primers. Each sample comprised RNA isolated from 3–5 individual plants. At least three technical replicates were performed. An ABI System Sequence Detector (QuantStudio^TM^ 6 Flex real-time PCR system; Applied Biosystems, Foster City, CA, United States) was used with the following thermal cycling conditions: Stage 1, 1 cycle at 50 °C for 2 min; Stage 2, 1 cycle at 95 °C for 10 min; Stage 3, 40 cycles at 95 °C for 15 s, 60 °C for 1 min; dissociation stage, 15 s at 95 °C, 15 s at 60 °C, 15 s at 95 °C. After amplification, melting curve analysis was performed to verify the product. Measured Ct values were converted to relative copy numbers using the ∆∆*C*t method. Values were normalized by comparing them with *CmoActin* or *CsaActin*, for which primers are listed in Table S9.

Both RT-PCR and RT-qPCR primers recognizing specific sequence region of Csa/Cmo transcripts were designed using CLC Sequence Viewer 8 (QIAGEN Aarhus A/S) based on the published consensus sequences in the Cucumber Genome Database (http://www.icugi.org/).

### 4.11. Data Statistical Analysis

All the original statistical data of transcript abundance of genes in this study including mean value, standard deviation, and significance were firstly analyzed using EXCEL (Office 2010). The program was listed as: (1) EXCEL (Office 2010): AVERAGE ( ), SDEV ( ); (2) EXCEL (Office 2010): Student *T*-test ( ) (*p* < 0.001). Then, graphs of RT-qPCR were generated by GraphPad Prism5.0.

To analyze Principal Component Analysis (PCA) of mobile mRNAs in all different species, the information of mean CDS length of all genes were firstly extracted by using software SeqKit [49]. Percentage of mobile mRNAs were analyzed by EXCEL (Office 2010) as: Percentage of mobile mRNAs = Number of mobile mRNAs/Number of total gene; Percentage of nonmobile mRNAs = Number of nonmobile mRNAs/Number of total gene; Mean CDS length of mobile mRNAs = AVERAGE (mobile mRNAs); Mean CDS length of nonmobile mRNAs = AVERAGE (nonmobile mRNAs). Then, one EXCEL list including four parameters included percentage of mobile mRNAs, percentage of nonmobile mRNAs, CDS length of mobile mRNAs and CDS length of nonmobile mRNAs were uploaded in the Omicshare PCA tools (omicshare.com/tools).

### 4.12. Data Availability

All sequencing datasets are available at the NCBI Small Read Archive (SRA) under ID PRJNA552914. All accession numbers of related genes in this study are listed in Table S9.

## 5. Conclusions

In this study, the integration of transcriptome and genomic re-sequencing of a heterograft system with cucumber (*Cucumis sativu*s, Csa) and pumpkin (*Cucurbita moschata*, Cmo) was used to study mRNA exchanges between scion and rootstock. We found that 3923 Csa mRNAs and 1788 Cmo mRNAs systemically migrated through the allocation pathway from source to sink. Then, GO and KEGG analyses of mobile mRNAs in each direction for cucumber and pumpkin exhibited that mobile mRNAs can participate in plant growth and development processes both universally common and individually specific. In particular, Csa scion move-down mRNAs were involved in carbon fixation and biosynthesis of amino acid pathways. Cmo root received Csa move-down mRNA and then delivered the corresponding Cmo upward mRNA to scion to improve photosynthesis of the cucumber scion. This formed a feedback signal loop of scion-to-rootstock-to scion to explain how pumpkin rootstock enhanced cucumber production in the industry. Cmo move-down mRNA were mainly related to sulfur metabolism, thus failing to exchange carbon metabolism signals to Csa rootstock. It was a single direction signal pathway between Cmo scion and Csa rootstock. Finally, RT-PCR, RT-qPCR, and colony sequencing were used to estimate the mobility of 78.75% of selected mobile transcripts.

## Supplementary Materials

The following are available online at https://www.mdpi.com/1422-0067/21/15/5253/s1, Figure S1. PCR verification of harvested material using specific primers. Genotype contamination verification of harvested material using genomic PCR with primers recognizing both CsaACTIN7 and CmoACTIN7. RT-PCR with specific primers recognizing the well-known mobile mRNA Cmo/CsaNACP was performed as a mobility positive control. gDNA, genomic DNA; gPCR, genomic PCR; Csa, Cucumis sativus; Cmo, Cucurbita moschata; S, shoot; R, root; Neg, negative. Figure S2. Analysis on Csa/Cmo mRNA mobility by RT-PCR product colony sequencing. Partial sequencing results confirm that mobile Csa/Cmo mRNA originated from heterologous tissue. For example, the sequence of the *CmoCaBP* RT-PCR product amplified from Cmo/Csa root was similar to that from Cmo/Cmo root tissue, demonstrating that *CmoCaBP* mRNA indeed moved from Cmo scion to Csa root. Figure S3. Real-time amplification curve and product dissolution curve of *CmoPHO1* and *CsaADH*. ABI System Sequence Detector (QuantStudio^TM^ 6 Flex real-time PCR system; Applied Biosystems) was used with the following thermal cycling conditions. Stage 1, one cycle at 50 °C for 2 min. Stage 2, one cycle at 95 °C for 10 min; Stage 3, 40 cycles at 95 °C for 15 s and 60 °C for 1 min; Dissociation stage, 15 s at 95 °C, 15 s at 60 °C, and 15 s at 95 °C. Table S1. Mobile Csa (3923) and Cmo (1788) mRNAs detected in heterografts. Table S2. Overlapping mobile Csa and Cmo mRNAs and orthologous mRNAs in the same direction. Table S3. Characterization of 18 mobile mRNAs shared in different heterograft systems. Table S4. Orthologous mobile mRNAs identified in different heterograft systems. Table S5. Top 20 GO enrichment categories of Csa and Cmo mRNAs in different directions. Table S6. KEGG enrichment of mobile Csa and Cmo mRNAs in different directions. Table S7. Identification of mobile Csa and Cmo mRNAs in heterografts by PCR. Table S8. Original ct value of *CmoPHO1* and *CsaADH.* Table S9. Oligonucleotides used in the study. Table S10. Four parameters of mobile mRNA were used for PCA analysis in six species.
