# Supplementary material for: Identification of Long-Distance Transmissible mRNA between Scion and Rootstock in Cucurbit Seedling Heterografts
Source: Int J Mol Sci. 2020 Jul 24;21(15):5253. doi: 10.3390/ijms21155253 (PMC7432352; doi:10.3390/ijms21155253)

Supplemental figure 2
Liu et al., 2020

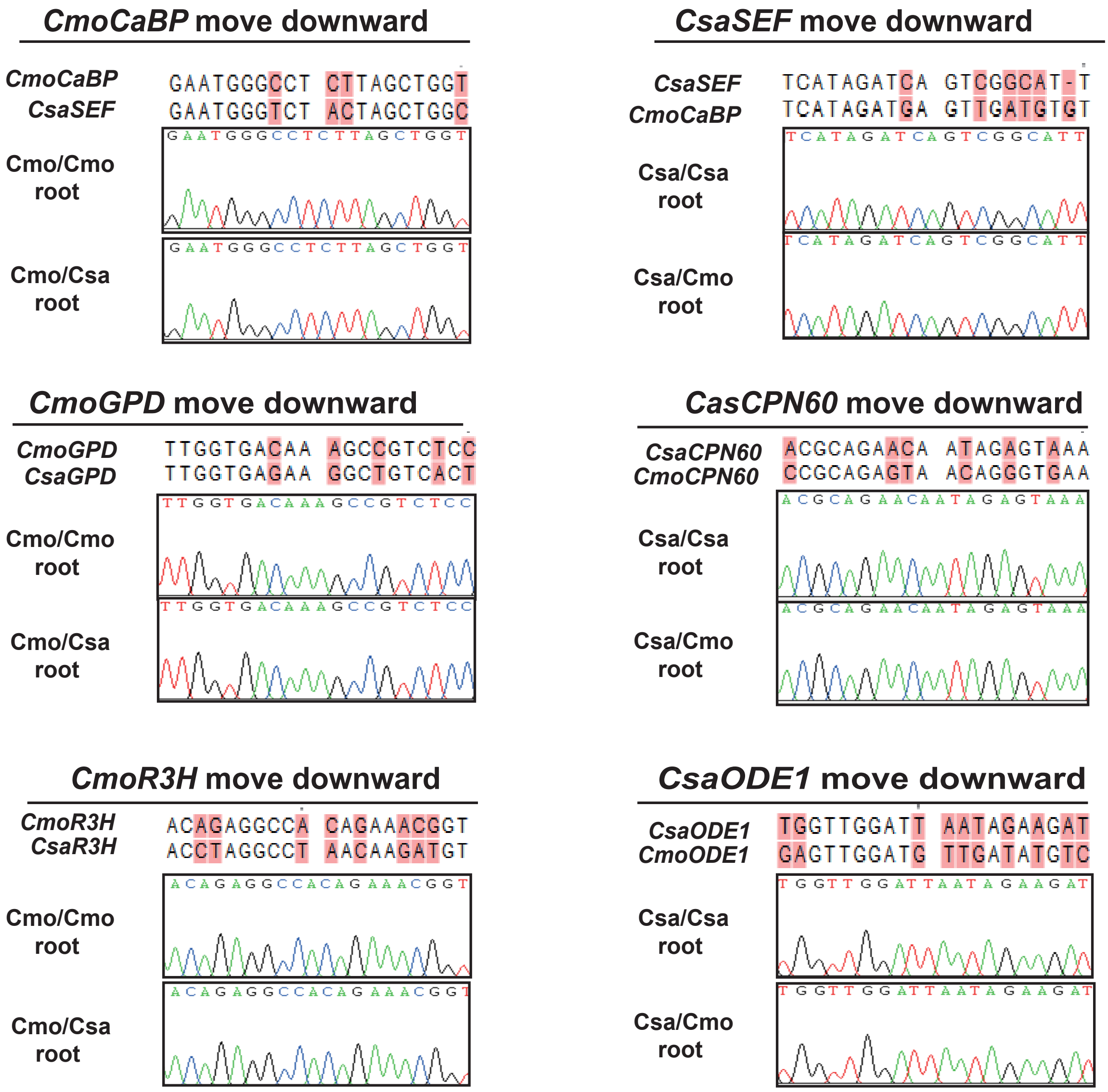

Supplement: Supplementary file 1 [file ijms-21-05253-s001.zip › Supplementary files/Supplementary Figure S2.pdf]
