# Supplementary material for: Identification of Long-Distance Transmissible mRNA between Scion and Rootstock in Cucurbit Seedling Heterografts
Source: Int J Mol Sci. 2020 Jul 24;21(15):5253. doi: 10.3390/ijms21155253 (PMC7432352; doi:10.3390/ijms21155253)
Supplement: Supplementary file 1 [file ijms-21-05253-s001.zip › Supplementary files/Supplementary Figure S3.pdf]

Supplemental figure 3

Liu et al., 2020

CmoPHO1

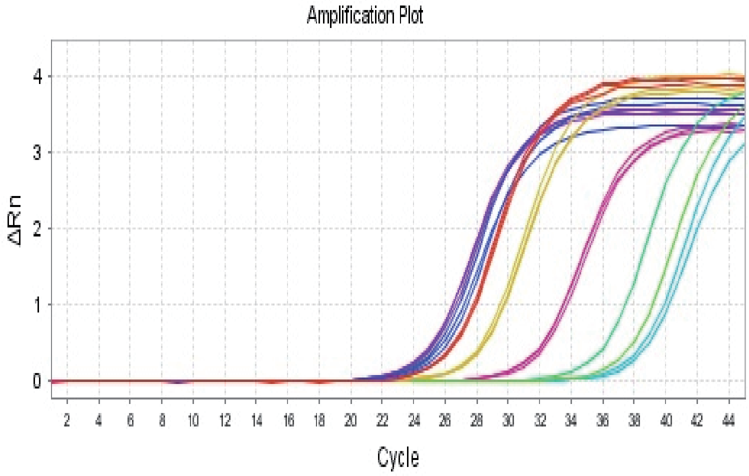

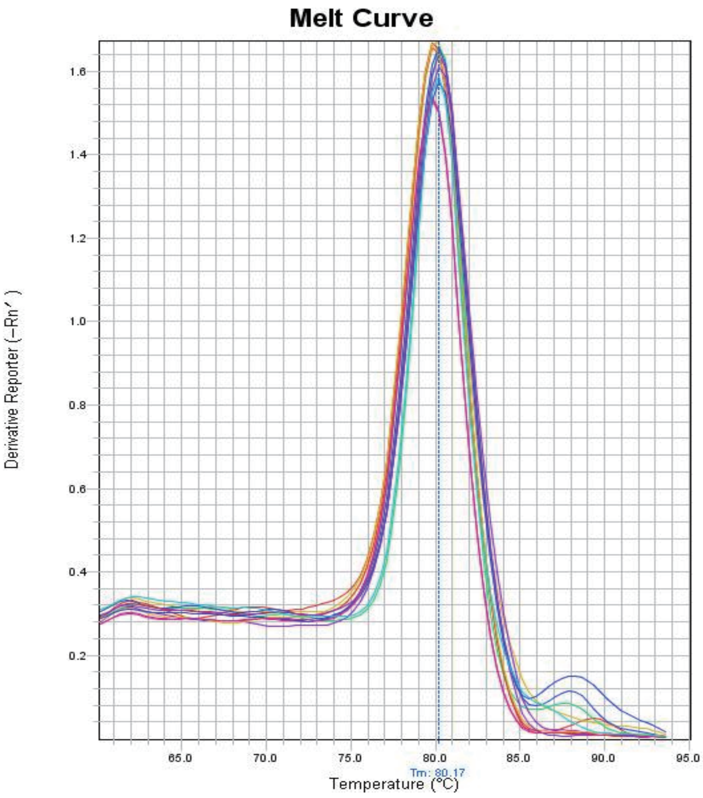

CsaADH

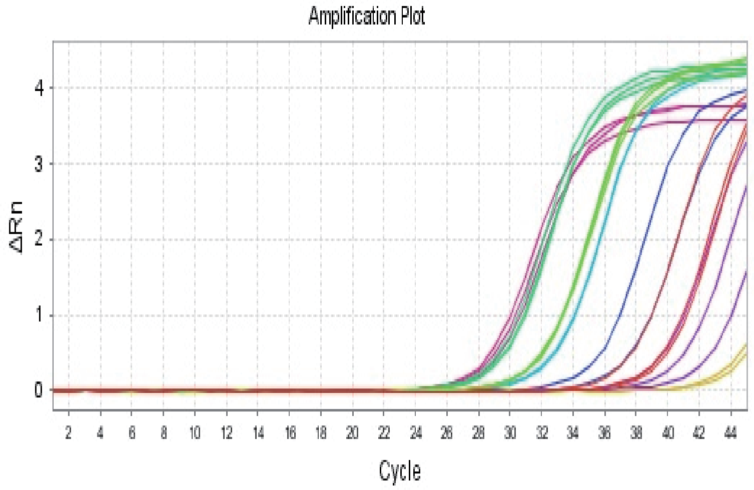

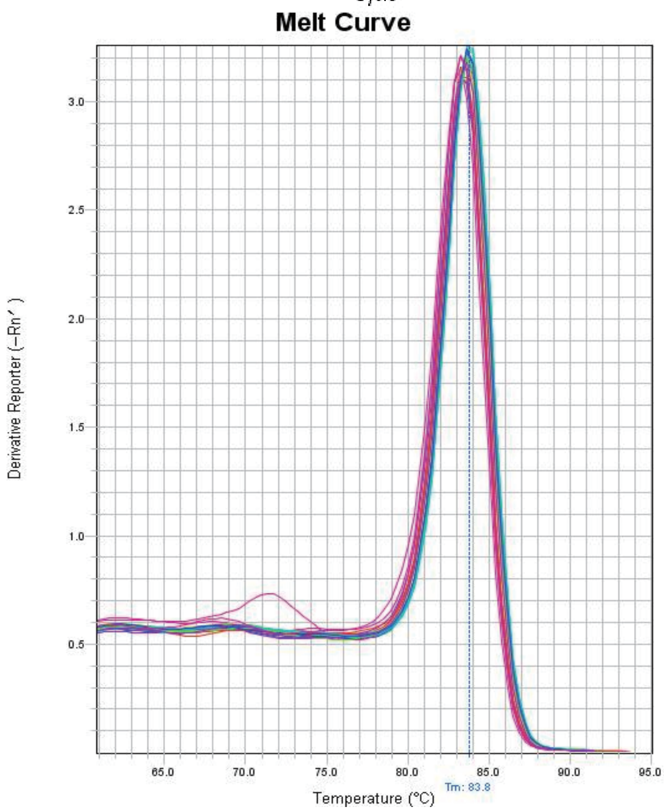

Sample name	Original ct value of *CmoPHO1*	
	Leaf	Root
Cmo/Cmo	26.4669075	28.3325882
	26.56741333	28.46439934
	26.61351204	28.53124237
Csa/Csa	37.74569823	36.38644028
	37.80557251	36.26458274
	37.97047806	36.19257736
Csa/Cmo	38.98477402	25.69463501
	38.96075058	25.77748032
	38.94798527	25.84656143
Cmo/Csa	25.16706352	32.14624176
	25.19011879	32.18393707
	25.17412376	32.18128967

Sample name	Original ct value of *CsaADH*	
	Leaf	Root
Cmo/Cmo	39.88830795	44.42795181
	39.78833389	44.52412415
	39.69978333	44.67031784
Csa/Csa	32.09522629	29.71139717
	32.04417419	29.69691467
	32.08501396	29.51380653
Csa/Cmo	32.83490448	35.96512222
	32.82249069	35.87364769
	32.86444855	35.73124313
Cmo/Csa	39.73345184	29.03825722
	39.76423569	29.04762268
	39.74823761	29.09628754
