# Supplementary material for: Identification of Long-Distance Transmissible mRNA between Scion and Rootstock in Cucurbit Seedling Heterografts
Source: Int J Mol Sci. 2020 Jul 24;21(15):5253. doi: 10.3390/ijms21155253 (PMC7432352; doi:10.3390/ijms21155253)
Supplement: Supplementary file 1 [file ijms-21-05253-s001.zip › Supplementary files/Table S9 .docx]

**Supplementary Table S9 Oligonucleotides used in the study**

| **Gene ID** |  | **Name and sequence** | **Size of RT-PCR fragment** | **Purpose** |
| --- | --- | --- | --- | --- |
| CmoCh02G006200/ CsGy2G015500 | *Cmo/CsaACTIN7* | ACTIN-F 5’- ATGAGCTTCGTGTTGCACCT-3' | 576 bp | common primer for RT-PCR |
|  |  | ACTIN-R 5’- TCACACTTCATGATTGAGTTGTAGG-3' |  |  |
| CmoCh02G006200 | *CmoACTIN7* | CmoACTIN-F 5’- GATTAAAATCGTGCAGCTTACAGT-3' | 226 bp | *CmoACTIN* primer for pollution identification |
|  |  | CmoACTIN-R 5’- TGTGCTATGCACATTGAACGG-3' |  |  |
| CsGy2G015500 | *CsaACTIN7* | CsaActin-F 5’- TTCTTTGATTACAATCGTGTTGCTT-3' | 204 bp | *CsaACTIN* primer for pollution identification |
|  |  | CsaActin-R 5’- ACACTTGCCAAACAATCACACTAA-3' |  |  |
| CmoCh02G006200 | *CmoACTIN7* | CmoActin-F 5’-TGCCAATCTACGAGGGTTATG-3' | 159 bp | Internal reference for qRT-PCR |
|  |  | CmoActin-R 5’-GCTTTTCTTTCATGTCACGGA-3' |  |  |
| CsGy2G015500 | *CsaACTIN7* | CsaActin-F 5’-CTGGTATCGCTGACCGTATG-3' | 196 bp | Internal reference for qRT-PCR |
|  |  | CsaActin-R 5’-CTGTGGACGATGGATGGG-3' |  |  |
| CmoCh01G014300 | *CmoNAC2* | CmoNAC2-F 5’-CTCCGAGCAGGTGGTGTC-3' | 108 bp | primer for qRT-PCR |
|  |  | CmoNAC2-R 5’-TCCAGTGAAGCGTCCAGGT-3' |  |  |
| CmoCh13G007620 | *CmoRBP45* | CmoRBP45-F 5’-CATGGGGCCGTAGTCCTT-3' | 201 bp | primer for qRT-PCR |
|  |  | CmoRBP45-R 5’-GCGGTCCTGGTTGTTGGTA-3' |  |  |
| CmoCh04G017560 | *CmoPHO1* | CmoPHO1-F 5’-CAAGCCTACATGGATTACGAGTT-3' | 98 bp | primer for qRT-PCR |
|  |  | CmoPHO1-R 5’-GCAGAGTCGGTTAAGGGGTG-3' |  |  |
| CmoCh08G001830 | *CmoHIK3* | CmoHIK3-F 5’-CCCGCCATTGTCACCAG-3' | 149 bp | primer for qRT-PCR |
|  |  | CmoHIK3-R 5’-CGATACCCGTTCCTCCGT-3' |  |  |
| CmoCh02G016880 | *CmoARH* | CmoARH-F 5’-TGGTGACAAAGCACAAACTGAACG-3' | 101 bp | primer for qRT-PCR |
|  |  | CmoARH-R 5’-CTAAACCTCGGGCGGCTACAT-3' |  |  |
| CmoCh17G008350 | *CmoUNP* | CmoUNP-F 5’-CACTGTCCTCCATCATATCCTGT-3' | 111 bp | primer for qRT-PCR |
|  |  | CmoUNP-R 5’-CTTCTTTTTCTGAGCTTTGGGT-3' |  |  |
| CmoCh02G017480 | *CmoTFP18* | CmoTFP18-F 5’-TCTCGTGGAAGTGGCTTTGGT-3' | 170 bp | primer for qRT-PCR |
|  |  | CmoTFP18-R 5’-GAGACAATGCCTGCGTTGAAAT-3' |  |  |
| CmoCh11G019300 | *CmoTHF1* | CmoTHF1-F 5’-CAGTTCTCGCTTGGTCATTCA-3' | 107 bp | primer for qRT-PCR |
|  |  | CmoTHF1-R 5’-TGCTGGGGATAGGTCGTTTAT-3' |  |  |
| CmoCh09G004630 | *CmoCAF* | CmoCAF-F 5’-ATTGGGAGTTGGCTTTGGT-3' | 284 bp | primer for qRT-PCR |
|  |  | CmoCAF-R 5’-TGATGGTGGTGGAACGCT-3' |  |  |
| CmoCh08G009130 | *CmoURT1* | CmoURT1-F 5’-AAGAGAGCAAAAGATTGGACG-3' | 180 bp | primer for qRT-PCR |
|  |  | CmoURT1-R 5’-GCACGGGTTTGGATAGGTT-3' |  |  |
| CmoCh01G001460 | *CmoPPD* | CmoPPD-F 5’-GCCGTTGGGACAATGTTTACT-3' | 268 bp | primer for qRT-PCR |
|  |  | CmoPPD-R 5’-CTTCAAGGCGGGAATGGATAT-3' |  |  |
| CmoCh01G018680 | *CmoPIP2* | CmoPIP2-F 5’-TCTTGGCATTCACGCTCTTC-3' | 131 bp | primer for qRT-PCR |
|  |  | CmoPIP2-R 5’-CAACGGTTCCCTCCTCTGT-3' |  |  |
| CmoCh14G007690 | *CmoASF2* | CmoASF2-F 5’-GCCTGGCTTCCATTTATCC-3' | 258 bp | primer for qRT-PCR |
|  |  | CmoASF2-R 5’-CGACCAATCCTTCTTCCGT-3' |  |  |
| CmoCh00G000020 | *CmoRPL26* | CmoRPL26-F 5’-CGAAGGTAAGGTCGTACAGGT-3' | 223 bp | primer for qRT-PCR |
|  |  | CmoRPL26-R 5’-CTCAGCGGTGAATTTAGTGC-3' |  |  |
| CmoCh17G004180 | *CmoCYS* | CmoCYS-F 5’-GAAGGACTAATGGTCGGGATA-3' | 184 bp | primer for qRT-PCR |
|  |  | CmoCYS-R 5’-CTGAGACGGGTTGCATGTTC-3' |  |  |
| CmoCh08G003690 | *CmoGID1B* | CmoGID1B-F 5’-AACCGAATCCGAGAAGAAAC-3' | 159 bp | primer for qRT-PCR |
|  |  | CmoGID1B-R 5’-GGGAAGTCGAGTCCTGCTATG-3' |  |  |
| CmoCh13G007660 | *CmoPEX* | CmoPEX-F 5’-GTGCGTCTTTCAGGTATGGTT-3' | 163 bp | primer for qRT-PCR |
|  |  | CmoPEX-R 5’-GTTCTTGTGGCGCTTGTAGAG-3' |  |  |
| CmoCh02G009300 | *CmoARF* | CmoARF-F 5’-TCACTAAGGGTTCAATGGGATG-3' | 149 bp | primer for qRT-PCR |
|  |  | CmoARF-R 5’-GTTTCAGTTGATTGTCGAGGC-3' |  |  |
| CmoCh05G012620 | *CmoSPK* | CmoSPK-F 5’-TCGTGAAAGGTCGGTTGC-3' | 196 bp | primer for qRT-PCR |
|  |  | CmoSPK-R 5’-CCGTCTTGCTTCAAATGCT-3' |  |  |
| CmoCh11G019620 | *CmoSPE2C* | CmoSPE2C-F 5’-GAGTTTTGGAAGTCGCAGGA-3' | 141 bp | primer for qRT-PCR |
|  |  | CmoSPE2C-R 5’-GGAAGTACCGCACTCGCAT-3' |  |  |
| CmoCh02G006200 | *CmoACT7* | CmoACT7-F 5’-TGCCAATCTACGAGGGTTATG-3' | 267 bp | primer for qRT-PCR |
|  |  | CmoACT7-R 5’-CTCCGATTGTGATGACTTGC-3' |  |  |
| CmoCh04G023800 | *CmoGLD* | CmoGLD-F 5’-CATCATGGCCGCCATTAACAA-3' | 142 bp | primer for qRT-PCR |
|  |  | CmoGLD-R 5’-TCCAACTCCCACCGCCGTCTC-3' |  |  |
| CmoCh06G017440 | *CmoTUA3* | CmoTUA3-F 5’-CTATCCGTTCCCGAAATCAC-3' | 194 bp | primer for qRT-PCR |
|  |  | CmoTUA3-R 5’-CCAGTAGGACACCAGTCAACAA-3' |  |  |
| CmoCh10G005630 | *CmoGSG6* | CmoGSG6-F 5’-CTGGGGTGGTTGGCATCTA-3' | 234 bp | primer for qRT-PCR |
|  |  | CmoGSG6-R 5’-GAGAAACGGGAAGTGCTGAAT-3' |  |  |
| CmoCh03G003880 | *CmoTUB7* | CmoTUB7-F 5’-CTGGAAACAACTGGGCTAAAG-3' | 224 bp | primer for qRT-PCR |
|  |  | CmoTUB7-R 5’-TGGAAAGACGGAGAAGGTAAG-3' |  |  |
| CmoCh06G014450 | *CmoGLS* | CmoGLS-F 5’-AGGTTGACAGGGAAGCACG-3' | 176 bp | primer for qRT-PCR |
|  |  | CmoGLS-R 5’-TCTGCCAGCAGCGAGGT-3' |  |  |
| CmoCh11G018220 | *CmoGPA* | CmoGPA-F 5’-GCGTCAAGCGGCAGTTC-3' | 208 bp | primer for qRT-PCR |
|  |  | CmoGPA-R 5’-CGTCTGGATCGAGGCAAGT-3' |  |  |
| CmoCh10G003180 | *CmoGRP2* | CmoGRP2-F 5’-GGAGGAGGTTTTGGGTCAG-3' | 297 bp | primer for qRT-PCR |
|  |  | CmoGRP2-R 5’-ATTCAGGGTGTTAGCTTCGTT-3' |  |  |
| CmoCh04G004280 | *CmoGBP* | CmoGBP-F 5’-TAGGCACCAGGGGTTCAG-3' | 192 bp | primer for qRT-PCR |
|  |  | CmoGBP-R 5’-AACCTGCGGTGGGACG-3' |  |  |
| CmoCh01G009430 | *CmoCES* | CmoCES-F 5’-ATCACAGCGAACACCGTCC-3' | 206 bp | primer for qRT-PCR |
|  |  | CmoCES-R 5’-TCAACCTTCAGGGCAAAATAC-3' |  |  |
| CmoCh07G006010 | *CmoARP* | CmoARP-F 5’-CATGGAAGGGTACTCAGAGGTG-3' | 276 bp | primer for qRT-PCR |
|  |  | CmoARP-R 5’-GGGGCGTTATTGTGGTCAT-3' |  |  |
| CsGy2G016100 | *CsaNAC2* | CsaNAC2-F 5’-CAACGACATGGTACAACTCCCT-3' | 148 bp | primer for qRT-PCR |
|  |  | CsaNAC2-R 5’-GGATTGACTTTGGACCTCCTTAT-3' |  |  |
| CsGy1G011590 | *CsaSUT* | CsaSUT-F 5’-GGCTTTGCCAATCAGTCAG-3' | 178 bp | primer for qRT-PCR |
|  |  | CsaSUT-R 5’-CACCTAAACTCAAACGCCATC-3' |  |  |
| CsGy3G021710 | *CsaHSP16* | CsaHSP16-F 5’-GGATGGAGACGATGAGACAGA-3' | 186 bp | primer for qRT-PCR |
|  |  | CsaHSP16-R 5’-AATGCTCGAACTTGAGGTGAA-3' |  |  |
| CsGy5G015960 | *CsaPHO1* | CsaPHO1-F 5’-GCGTTATCGGCTTCTACTCCTA-3' | 124 bp | primer for qRT-PCR |
|  |  | CsaPHO1-R 5’-CCCCATCTTCATTCAACTCATC-3' |  |  |
| CsGy5G022340 | *CsaTPS6* | CsaTPS6-F 5’-ATCTTCGCCTGTACTGTTGGA-3' | 136 bp | primer for qRT-PCR |
|  |  | CsaTPS6-R 5’-CACGCATCTGTGGTAGTTGTC-3' |  |  |
| CsGy6G007930 | *CsaHIK3* | CsaHIK3-F 5’-GTTATCCTGTTGCCAATAGACG-3' | 231 bp | primer for qRT-PCR |
|  |  | CsaHIK3-R 5’-TCAGCCCGATACCCGTTC-3' |  |  |
| CsGy3G026070 | *CsaARH* | CsaARH-F 5’-CCTACGAGGGAATTAGCTCAAC-3' | 184 bp | primer for qRT-PCR |
|  |  | CsaARH-R 5’-GGACCACAAAGGATACTTCTGC-3' |  |  |
| CsGy7G012060 | *CsaADH* | CsaADH-F 5’-CCACAAGCTAATGAAGTCCGT-3' | 229 bp | primer for qRT-PCR |
|  |  | CsaADH-R 5’-CCGATCTACAATGGCGACA-3' |  |  |
| CsGy7G009360 | *CsaUNP* | CsaUNP-F 5’-AACAAATTCCCTCTTCAACTCATCT-3' | 225 bp | primer for qRT-PCR |
|  |  | CsaUNP-R 5’-AGTAACTGCTCCTTCATCGAGAAGA-3' |  |  |
| CsGy3G025280 | *CsaTFP18* | CsaTFP18-F 5’-CGGCTAGTTCGGTTTCGTTTT-3' | 158 bp | primer for qRT-PCR |
|  |  | CsaTFP18-R 5’-GATGAACCAAGGGGCTGGAAG-3' |  |  |
| CsGy7G003860 | *CsaTHF1* | CsaTHF1-F 5’-TGTTCCGATAGGAGATTGCTG-3' | 127 bp | primer for qRT-PCR |
|  |  | CsaTHF1-R 5’-TGCGAGAACTGAAGGTGGAT-3' |  |  |
| CsGy3G041420 | *CsaCAF* | CsaCAF-F 5’-AGCTAATGGTGCAAACGGAT-3' | 159 bp | primer for qRT-PCR |
|  |  | CsaCAF-R 5’-AGTGCTGCTTTCTGCCTTG-3' |  |  |
| CsGy6G005680 | *CsaURT1* | CsaURT1-F 5’-AGGTAAAGGCTTCGGAGGTAT-3' | 136 bp | primer for qRT-PCR |
|  |  | CsaURT1-R 5’-TCACCAAATTGACTTGAACGAG-3' |  |  |
| CsGy3G038250 | *CsaTCP1* | CsaTCP1-F 5’-TAGGATGGGTCTACACCCTAG-3' | 101 bp | primer for qRT-PCR |
|  |  | CsaTCP1-R 5’-TCTCCGAACCTTTCTCCACTA-3' |  |  |
| CsGy4G007640 | *CsaMPK* | CsaMPK-F 5’-AAACACTTCTCACTGCCACG-3' | 170 bp | primer for qRT-PCR |
|  |  | CsaMPK-R 5’-ACTCGTCCAGTAGGCACTTTAG-3' |  |  |
| CsGy4G022750 | *CsaPIP2* | CsaPIP2-F 5’-GCCAAAGAAGCCAACGGTA-3' | 226 bp | primer for qRT-PCR |
|  |  | CsaPIP2-R 5’-TCAACGGCGAGTCTCCAA-3' |  |  |
| CsGy3G024470 | *CsaASF2* | CsaASF2-F 5’-GGCTGGTAATGTTGCTGTCA-3' | 176 bp | primer for qRT-PCR |
|  |  | CsaASF2-R 5’-GTTTTGTGGGGTCGAGGA-3' |  |  |
| CsGy2G014100 | *CsaRPL26* | CsaRPL26-F 5’-CAGGTCTACCGTCGCAAGTG-3' | 160 bp | primer for qRT-PCR |
|  |  | CsaRPL26-R 5’-CCTTAGCCTTACGATCAAGCA-3' |  |  |
| CsGy6G000650 | *CsaCYS* | CsaCYS-F 5’-AAGGAAGGACTAATGGTGGGA-3' | 180 bp | primer for qRT-PCR |
|  |  | CsaCYS-R 5’-TGGTTGCATGTTCTCAGCTTC-3' |  |  |
| CsGy7G015120 | *CsaGID1B* | CsaGID1B-F 5’-TCATCAGCCAACAGTGCTATCT-3' | 220 bp | primer for qRT-PCR |
|  |  | CsaGID1B-R 5’-CAGAACTGTCTCCAGCCAAAT-3' |  |  |
| CsGy1G010600 | *CsaPEX* | CsaPEX-F 5’-GCTGTACCCAGAAGTAGACCCT-3' | 187 bp | primer for qRT-PCR |
|  |  | CsaPEX-R 5’-ATCCACAATCAGCAGTCCCT-3' |  |  |
| CsGy4G006490 | *CsaARF* | CsaARF-F 5’-GGGCAAGCCAGTTTATCGT-3' | 282 bp | primer for qRT-PCR |
|  |  | CsaARF-R 5’-GCGGAATGCTCGGTGTAG-3' |  |  |
| CsGy1G017750 | *CsaSPK* | CsaSPK-F 5’-GGCAGGGAAATTCAGCGT-3' | 134 bp | primer for qRT-PCR |
|  |  | CsaSPK-R 5’-TCACCTCCACCACCAGAAAC-3' |  |  |
| CsGy1G012170 | *CsaSPE2C* | CsaSPE2C-F 5’-GCTACGATAGTGCCGTTTGC-3' | 179 bp | primer for qRT-PCR |
|  |  | CsaSPE2C-R 5’-TCGGTTGTACTCCTCGCTTC-3' |  |  |
| CsGy4G015910 | *CsaPTA* | CsaPTA-F 5’-GGAAAGAAGACTTGACTGATAAACC-3' | 237 bp | primer for qRT-PCR |
|  |  | CsaPTA-R 5’-GTCCACATATACCAACACCCAC-3' |  |  |
| CsGy5G013610 | *CsaGLD* | CsaGLD-F 5’-CGGTTCAACATCGTGTCAAA-3' | 139 bp | primer for qRT-PCR |
|  |  | CsaGLD-R 5’-GAGGCATGGTGTAAGCTGGTA-3' |  |  |
| CsGy3G038150 | *CsaTUA3* | CsaTUA3-F 5’-TACAGTGGGCAGGGAAATAGT-3' | 161 bp | primer for qRT-PCR |
|  |  | CsaTUA3-R 5’-CGTAATCCACAGACAAGCGTT-3' |  |  |
| CsGy1G017020 | *CsaGSG6* | CsaGSG6-F 5’-TGGCGACCTCGTCACTCTT-3' | 257 bp | primer for qRT-PCR |
|  |  | CsaGSG6-R 5’-GATTTTCCACCCGCTCACT-3' |  |  |
| CsGy1G024280 | *CsaTUB7* | CsaTUB7-F 5’-AACAATACCGAGCCCTTACAG-3' | 260 bp | primer for qRT-PCR |
|  |  | CsaTUB7-R 5’-TGATGCCATTTTCAGACCCT-3' |  |  |
| CsGy3G013760 | *CsaGLS* | CsaGLS-F 5’-TCATCACCGTCATCGCTTCGT-3' | 166 bp | primer for qRT-PCR |
|  |  | CsaGLS-R 5’-CCCATAACCCCAGCAATCAAA-3' |  |  |
| CsGy7G002150 | *CsaGPAT* | CsaGPAT-F 5’-GCAAGATTCACCACTCTGTCG-3' | 299 bp | primer for qRT-PCR |
|  |  | CsaGPAT-R 5’-CTTGATAACTGTCACAATCCCAC-3' |  |  |
| CsGy4G009180 | *CsaGBP* | CsaG BP-F 5’-TCCCTTCTACAACACCCATTTC-3' | 158 bp | primer for qRT-PCR |
|  |  | CsaG BP-R 5’-GACGGAGCGGTTGGTAGTTAG-3' |  |  |
| CsGy6G002430 | *CsaCES* | CsaCES-F 5’-GAGCTTAGCAACTATGCCAGTATT-3' | 187 bp | primer for qRT-PCR |
|  |  | CsaCES-R 5’-CCAACACCTTTAGCAGACCCT-3' |  |  |
| CsGy2G021610 | *CsaARP* | CsaARP-F 5’-AGGCTACAGAACTGAGGCTTG-3' | 279 bp | primer for qRT-PCR |
|  |  | CsaARP-R 5’-CTTTCCCCATTTGACTTCCA-3' |  |  |
| CmoCh13G008180 | *CmoGPD* | CmoGPD-F 5'-TTCACTCAATCACTGCCACAC-3' | 186 bp | primer for qRT-PCR |
|  |  | CmoGPD-R 5'-CATCGACGGTAGGAACACG-3' |  |  |
| CmoCh01G003130 | *CmoRPL19* | CmoRPL19-F 5'-GCGTGTCCTGAGGAGATTACT-3' | 227 bp | primer for qRT-PCR |
|  |  | CmoRPL19-F 5'-CATGCTTTCTTTCCCTACTGG-3' |  |  |
| CmoCh04G015010 | *CmoR3H* | CmoR3H-F 5'-GCCACAGAAACGGTCTCAAG-3' | 206 bp | primer for qRT-PCR |
|  |  | CmoR3H-R 5'-CACCCAAGGAACCACGATA-3' |  |  |
| CmoCh11G011110 | *CmoVPASa* | CmoVPASa-F 5'-GGAATATCATGGCGAGGAAG-3' | 221 bp | primer for qRT-PCR |
|  |  | CmoVPASa-R 5'-CCGAAAATAAGTAGGTGGTGCT-3' |  |  |
| CmoCh10G010090 | *CmoUPA* | CmoUPA-F 5'-TTAGTTGGAGTGTCACCTTATGC-3' | 179 bp | primer for qRT-PCR |
|  |  | CmoUPA-R 5'-CCATCTGCAAACCGACTGT-3' |  |  |
| CmoCh16G000640 | *CmoGS2* | CmoGS2-F 5'-GGCAGAAGATTGAACTAAAGGAC-3' | 142 bp | primer for qRT-PCR |
|  |  | CmoGS2-R 5'-AAGGGAGCGAGTAAACCATG-3' |  |  |
| CsGy4G021460 | *CsaSEF* | CsaSEF-F 5'-AGGTCGTAGAGGGCGTCG-3' | 259 bp | primer for qRT-PCR |
| CsGy4G021460 | *CsaSEF* | CsaSEF-R 5'-TCGGAAAATGTTAATGAGGCA-3'  CsaSEF-F 5'-TCTGCACCAGAGGAAGAGTC-3'  CsaSEF-R 5'-TCATCGCTCTGTTCCCCAAC-3' | 996 bp | primer for RT-PCR |
| CsGy1G009330 | *CsaGPD* | CsaGPD-F 5'-TAAGGCAGCCATCAAGGAAG-3' | 227 bp | primer for qRT-PCR |
|  |  | CsaGPD-R 5'-TGATTGTCCACATTGCCTCTG-3' |  |  |
| CsGy5G006890 | *CsaODE1* | CsaODE1-F 5'-CTTTGCCCCTTTCCTTATGAC-3' | 197 bp | primer for qRT-PCR |
|  |  | CsaODE1-R 5'-GAGGCTGACGGAGCACGA-3' |  |  |
| CsGy3G035030 | *CsaR3H* | CsaR3H-F 5'-ATTCGGGTTATTCGCTCG-3' | 191 bp | primer for qRT-PCR |
|  |  | CsaR3H-R 5'-AGAATGCCTGAACTGGTTGTAG-3' |  |  |
| CsGy2G009490 | *CsaAC* | CsaAC-F 5'-TCCCTCCACATGACCATCC-3' | 137 bp | primer for qRT-PCR |
|  |  | CsaAC-R 5'-GCAGCACTGTCACGAGCAAT-3' |  |  |
| CsGy7G007080 | *CsaPBP* | CsaPBP-F 5'-GTTGTGGTGATGAGGGATGG-3' | 213 bp | primer for qRT-PCR |
|  |  | CsaPBP-R 5'-CTTGTCAGCGGCTTCTTTTAC-3' |  |  |
| CsGy1G028560 | *Csa5MT* | Csa5MT-F 5'-GCATCCCACATTGTTGGTTATC-3' | 269 bp | primer for qRT-PCR |
|  |  | Csa5MT-R 5'-AATCCAATCTCACCACCGTTC-3' |  |  |
| CsGy3G013550 | *CsaAIP* | CsaAIP-F 5'-ATCGTCTTTGACAAGTCCACC-3' | 149 bp | primer for qRT-PCR |
|  |  | CsaAIP-R 5'-GCAAGCGATTTCCCACAAG-3' |  |  |
| CsGy2G009450 | *CsaEF1A* | CsaEF1A -F 5'-CACGCTCTTCTTGCTTTCAC-3' | 165 bp | primer for qRT-PCR |
|  |  | CsaEF1A -R 5'-GAAGGGGATTTTGTCTGGGT-3' |  |  |
| CsGy4G021460 | *CsaSEF* | CsaSEF-F 5'-AAGCCGTGACAAGCGAAAAA-3' | 517 bp | primer for RT-PCR |
|  |  | CsaSEF-R 5'-TTGAGTTCACCATGACCCCC-3' |  |  |
| CmoCh01G012880 | *CmoCaBP* | CmoCaBP-F 5'-TTGCGGCTTAATTCGTTGGAA-3' | 410 bp | primer for RT-PCR |
|  |  | CmoCaBP-R 5'-AAATCTATGGCAACTGAATCCTGAA-3' |  |  |
| CsGy2G009380 | *CsaUBC8* | CsaUBC8-F 5'-CAGAAGGAGGATATAAGTTGGGGA-3' | 406 bp | primer for RT-PCR |
|  |  | CsaUBC8-R 5'-CATTGGTTGAGCCATGGGGA-3' |  |  |
| CmoCh01G009670 | *CmoUBC8* | CmoUBC8-F 5'-TCTCCTTACGAGCAGACCCT-3' | 407 bp | primer for RT-PCR |
|  |  | CmoUBC8-R 5'-AGCTATCTCAGGCACCAACG-3' |  |  |
| CsGy6G011830 | *CsaCPN60* | CsaCPN60-F 5'-GGAGCTTAAACCCCCTGACC-3' | 452 bp | primer for RT-PCR |
| CsGy6G011830 | *CsaCPN60* | CsaCPN60-R 5'-GAGGCAGTTGTCGTCCCATC-3'  CsaCPN60-F 5'- GGTTGATGATTGCCGATGCC-3'  CsaCPN60-R 5'-GTCTCGCTGTTTCCGATTGA-3' | 1012 bp | primer for RT-PCR |
| CsGy1G009330 | *CsaGPD* | CsaGPD-F 5'-CTGCTTGATGGTGTGGTCGT-3' | 534 bp | primer for RT-PCR |
|  |  | CsaGPD-R 5'-TTTGGTAGGGACAGGGGAGA-3' |  |  |
| CmoCh13G008180 | *CmoGPD* | CmoGPD-F 5'-CCATATTCAGCTCTCGCCCAA-3' | 410 bp | primer for RT-PCR |
| CmoCh13G008180 | *CmoGPD* | CmoGPD-R 5'-CAGCAGCTTTCTCTTTGTCGG-3'  CmoGPD-F 5'-CAGCAGCACGGACCATAGAT-3'  CmoGPD-R 5'-GCTCATAGCTGAGCCTCTGT-3' | 1196 bp | primer for RT-PCR |
| CsGy5G006890 | *CsaODE1* | CsaODE1-F 5'-TTCCAGCAATGTGTGATGCG-3' | 401 bp | primer for RT-PCR |
|  |  | CsaODE1-R 5'-AGTCAACAAAAGCATGATGATACA-3' |  |  |
| CmoCh04G002260 | *CmoODE1* | CmoODE1-F 5'-TGCTGAACCATCGAGCTTTCT-3' | 426 bp | primer for RT-PCR |
|  |  | CmoODE1-R 5'-TCGTACGCATAACCCTGAAGT-3' |  |  |
| CsGy3G035030 | *CsaR3H* | CsaR3H-F 5'-TTGAGTTCCAGCATTTTCCTACTTC-3' | 462 bp | primer for RT-PCR |
|  |  | CsaR3H-R 5'-CCAATTCACCACCCAAGGTTC-3' |  |  |
| CmoCh04G015010 | *CmoR3H* | CmoR3H-F 5'-CCTGCTCGACAACAGCTAGA-3' | 434 bp | primer for RT-PCR |
|  |  | CmoR3H-R 5'-TCCTGTAGTCTAGGATCGGCA-3' |  |  |
| CmoCh01G010050 | *CmoCK1* | CmoCK1-F 5'-TCACAACCTTGACATGCCTGG-3' | 588 bp | primer for RT-PCR |
|  |  | CmoCK1-R 5'-GGAGAAGGTAAGAGGTGAGGC-3' |  |  |
| CsGy5G027230 | *CsaCK1* | CsaCK1-F 5'-GGCACATCAGTGAAAACAGGTG-3' | 458 bp | primer for RT-PCR |
|  |  | CsaCK1-R 5'-TTCGTGCAAAGAGTAAATGAAGCC-3' |  |  |
| CmoCh03G002680 | *CmoNACP* | CmoNACP-F 5'-GATCAGTGGTGGATTCAATGGTG-3' | 510 bp | primer for RT-PCR |
|  |  | CmoNACP-R 5'-TCAACAAACCAAACCCATGAAAGT-3' |  |  |
| CsGy4G010940 | *CsaNACP* | CsaNACP-F 5'-CTATGGCAGCTACCACACCCAT-3' | 440 bp | primer for RT-PCR |
|  |  | CsaNACP-R 5'-CGACGATGCATCTTGGGTCA-3' |  |  |
| CmoCh11G000440 | *CmoASF* | CmoASF-F 5'-TGTACAAAGGCTCTGCGGC-3’ | 404 bp | primer for RT-PCR |
|  |  | CmoASF-R 5'-GCCAATGGTAGGAAGAGATGGT-3’ |  |  |
| CmoCh13G007920 | *CmoQS* | CmoQS-F 5'-AGTCAATCCATGCTAGTTTCTTCT-3’ | 402 bp | primer for RT-PCR |
|  |  | CmoQS-R 5'-TGCTAGCAGTGAGCTTGGAATG-3’ |  |  |
| CsGy1G000660 | *CsaASF* | CsaASF-F 5’-AATCCCTAACACTCGACCGTG-3’ | 395 bp | primer for RT-PCR |
|  |  | CsaASF-R 5’-TCCCATGTCCCCCAAACTTTTT-3’ |  |  |
| CsGy1G008820 | *CsaQS* | *CsaQS*-F 5’-CGAACTACCCAATAACAAAAGTGC-3’ | 410 bp | primer for RT-PCR |
|  |  | CsaQS-R 5’-CTCATCATCTTCAGCTAATGGATTT-3’ |  |  |
| CsGy1G005300 | *CsaGSTU9* | CsaGSTU9-F 5’-ATGCCAAGAGAGTCCAACTCG-3’ | 441 bp | primer for RT-PCR |
|  |  | CsaGSTU9-R 5’-TGTCCAAATATCCAAGTTCATCTCC-3’ |  |  |
| CmoCh06G014450 | *CmoGLS* | CmoGLS-F 5’-TGGTTGTAGGATCTTCGCTCT-3’ | 141 bp | primer for RT-PCR |
|  |  | CmoGLS0-R 5’-TTTCTTGCACAAGAGAAGTGC-3’ |  |  |
| CmoCh01G011840 | *CmoHSP105* | CmoHSP105-F 5’-GAACACGCTGATTCTCCCCA-3’ | 717 bp | primer for RT-PCR |
|  |  | CmoHSP105-R 5’-ATCAGAAGTGTGATGATTTGGTTC-3’ |  |  |
| CsGy1G025800 | *CsSRC2* | CsSRC2-F 5’-CTATTGTTCATCGTGAGGGCG-3’ | 148 bp | primer for RT-PCR |
|  |  | CsSRC2-R 5’-GGCAGCGACTCAATGTTATGT-3’ |  |  |
